# Modifiable Risk Factors Are Important Predictors of COVID-19-Related Mortality in Patients on Hemodialysis

**DOI:** 10.3389/fneph.2022.907959

**Published:** 2022-07-20

**Authors:** Jeroen Peter Kooman, Paola Carioni, Vratislava Kovarova, Otto Arkossy, Anke Winter, Yan Zhang, Francesco Bellocchio, Peter Kotanko, Hanjie Zhang, Len Usvyat, John Larkin, Stefano Stuard, Luca Neri

**Affiliations:** ^1^ Department of Internal Medicine, University Hospital Maastricht, Maastricht, Netherlands; ^2^ Fresenius Medical Care Italia SpA, Palazzo Pignano, Italy; ^3^ Fresenius Medical Care Deutschland GmbH, Bad Homburg, Germany; ^4^ Renal Research Institute, New York, NY, United States; ^5^ Icahn School of Medicine at Mount Sinai, New York, NY, United States; ^6^ Fresenius Medical Care, Waltham, MA, United States

**Keywords:** SARS-CoV-2, COVID-19, hemodialysis, chronic kidney disease, end-stage kidney disease, mortality, outcomes

## Abstract

**Introduction:**

Patients with end-stage kidney disease face a higher risk of severe outcomes from SARS-CoV-2 infection. Moreover, it is not well known to what extent potentially modifiable risk factors contribute to mortality risk. In this historical cohort study, we investigated the incidence and risk factors for 30-day mortality among hemodialysis patients with SARS-CoV-2 infection treated in the European Fresenius Medical Care NephroCare network using conventional and machine learning techniques.

**Methods:**

We included adult hemodialysis patients with the first documented SARS-CoV-2 infection between February 1, 2020, and March 31, 2021, registered in the clinical database. The index date for the analysis was the first SARS-CoV-2 suspicion date. Patients were followed for up to 30 days until April 30, 2021. Demographics, comorbidities, and various modifiable risk factors, expressed as continuous parameters and as key performance indicators (KPIs), were considered to tap multiple dimensions including hemodynamic control, nutritional state, and mineral metabolism in the 6 months before the index date. We used logistic regression (LR) and XGBoost models to assess risk factors for 30-day mortality.

**Results:**

We included 9,211 patients (age 65.4 ± 13.7 years, dialysis vintage 4.2 ± 3.7 years) eligible for the study. The 30-day mortality rate was 20.8%. In LR models, several potentially modifiable factors were associated with higher mortality: body mass index (BMI) 30–40 kg/m^2^ (OR: 1.28, CI: 1.10–1.50), single-pool Kt/V (OR off-target vs on-target: 1.19, CI: 1.02–1.38), overhydration (OR: 1.15, CI: 1.01–1.32), and both low (<2.5 mg/dl) and high (≥5.5 mg/dl) serum phosphate levels (OR: 1.52, CI: 1.07–2.16 and OR: 1.17, CI: 1.01–1.35). On-line hemodiafiltration was protective in the model using KPIs (OR: 0.86, CI: 0.76–0.97). SHapley Additive exPlanations analysis in XGBoost models shows a high influence on prediction for several modifiable factors as well, including inflammatory parameters, high BMI, and fluid overload. In both LR and XGBoost models, age, gender, and comorbidities were strongly associated with mortality.

**Conclusion:**

Both conventional and machine learning techniques showed that KPIs and modifiable risk factors in different dimensions ascertained 6 months before the COVID-19 suspicion date were associated with 30-day COVID-19-related mortality. Our results suggest that adequate dialysis and achieving KPI targets remain of major importance during the COVID-19 pandemic as well.

## Introduction

COVID-19 has greatly affected the patient population with end-stage kidney disease (ESKD). Patients on hemodialysis (HD) are more vulnerable to infection due to treatment with other patients in a confined space and shared transportation (1) and also at higher risk for adverse outcomes (2). Identifying patients at risk for adverse outcomes might aid individualized treatment decisions such as the need for hospitalization and more intensive monitoring. Even more importantly, the identification of modifiable risk factors for adverse outcomes might reduce the future mortality risk of COVID-19 in this population. Previous outcome studies in patients with Dialysis-Dependent Chronic Kidney Disease (CKD5D) identified predictive factors at the time of diagnosis (3, 4) when the disease might already have had a major impact on the parameters (5).

In dialysis therapy, various potentially modifiable risk factors have been identified that can also be expressed as key performance indicators (KPIs) for quality improvement purposes (6). To identify whether potentially modifiable risk factors or KPIs in the months before the COVID-19 suspicion date relate to outcome would be of significant clinical interest, as they could provide relevant intervention targets during a pandemic phase. In addition to conventional statistical models, machine learning (ML) is increasingly used in outcome prediction and diagnosis of COVID-19. In a recent study, we used a gradient descent (XGBoost) ML model for the early identification of patients with SARS-CoV-2 infection (7). The aim of the present study is to assess the association between potentially modifiable risk factors and KPIs, obtained before the onset of SARS-CoV-2 infection and 30-day COVID-19-related mortality, in HD patients. For this purpose, we applied both conventional and ML techniques.

## Materials and Methods

### Study Design, Consent, Patients, and Setting

**Figure 1:**
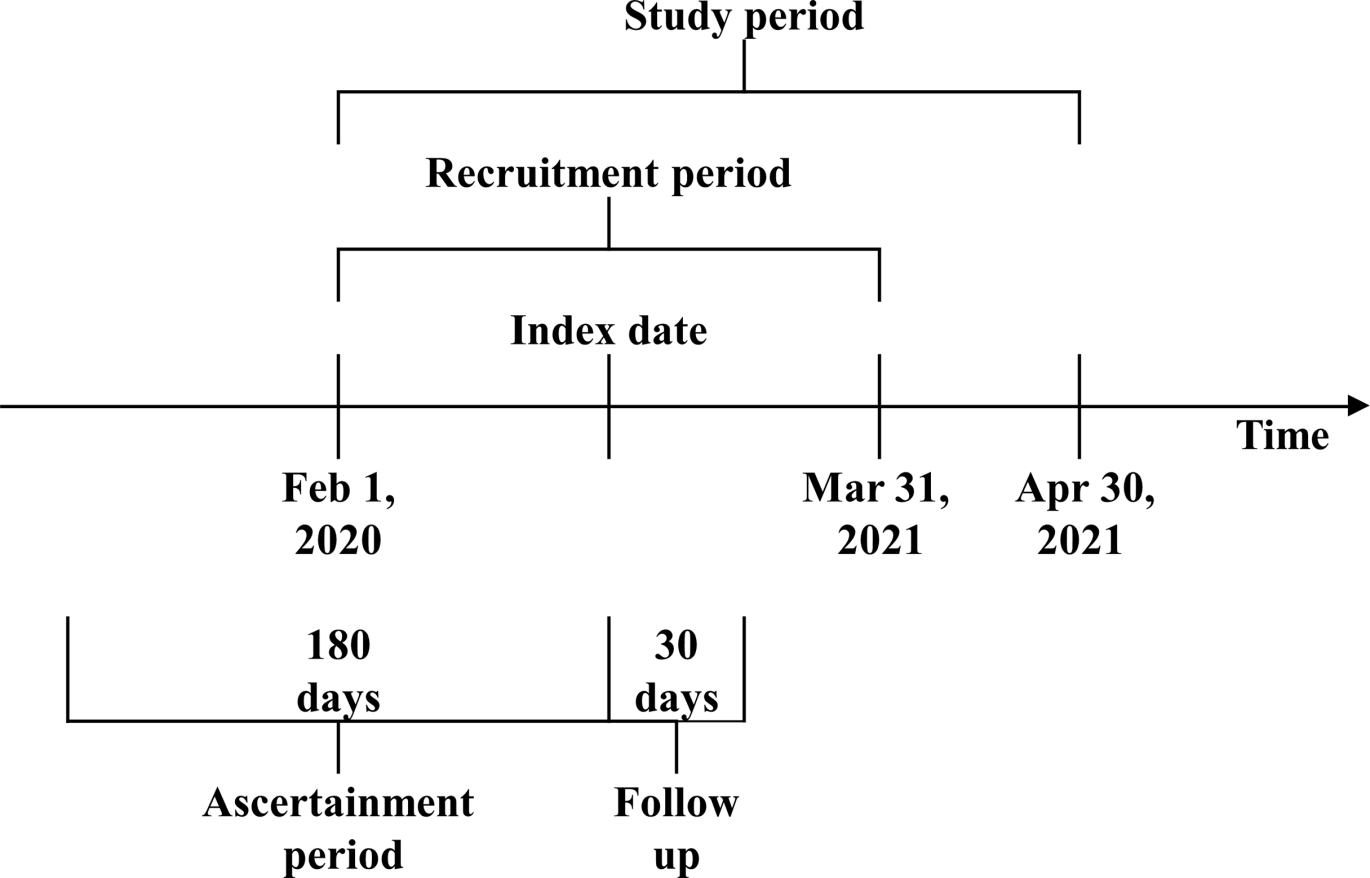
Study design.

A historical cohort study was conducted, evaluating 30-day mortality among HD patients with a documented SARS-CoV-2 infection (**Figure 1**). All adult patients treated in the European Fresenius Medical Care (FMC) NephroCare network from February 1, 2020, and March 31, 2021, who consented to their pseudo-anonymized data to be used for secondary data analysis, were screened for eligibility. All patients from countries that did not systematically report COVID-19 cases in the European Clinical Database (EuCliD^®^) (8, 9), namely, the United Kingdom, Ireland, Israel, Lebanon, and Switzerland, were excluded. COVID-19 cases were defined by the following priority algorithm:

1. SARS-CoV-2 infection confirmed by a documented PCR test. No restriction based on the PCR essay method adopted was imposed.

2. ICD10 codes suggestive of COVID-19 documented in the morbidity report: U07.1 and U07.2.

3. ICD10 codes suggestive of COVID-19 documented in the death reason report: U07.1 and U07.2.

For all patients with a documented SARS-CoV-2 infection, the index date for the study was the first suspicion date or the first occurrence of a suggestive ICD10 code. Patients with suspected SARS-CoV-2 infection and negative PCR assay were excluded from the study. In order to have a fully known health profile at the index date, the following were excluded: patients without renal replacement treatment onset date available, incident patients or patients who started dialysis more than 90 days before FMC registration, patients with no documented dialysis sessions in the 14 days before the index date, and patients ineligible for KPIs in the calendar month preceding the index date.

### Endpoint Definition

The primary endpoint was the occurrence of death within 30 days after the index date. Patients were considered alive at the end of the follow-up period only if recovered or with information about death or dialysis sessions after the end of the follow-up period.

### Measures

Demographic variables, anthropometric variables, lifestyle variables, comorbidities, COVID-19 vaccination status (at least one dose), treatment-related factors, and blood biomarkers in the 6-month ascertainment period (**Figure 1**) were assessed. Comorbidities were defined as any occurrence prior to the index date of suggestive ICD10 codes as described in **Supplementary Table S1**. Blood biomarkers and treatment-related variables were defined as the average in the 6-month ascertainment period. The achievement of key performance healthcare indicators (KPIs) was considered in the calendar month prior to the index date according to the operative definitions reported in **Supplementary Table S2**. Medication use at the index date was ascertained as the occurrence of suggestive ATC code as described in **Supplementary Table S3**.

### Data Management

A multivariate imputation by fully conditional specification regression method was applied on 31 continuous variables (SAS MI procedure) after a data-cleansing step considering missing any data that lie outside the listed upper or lower values as described in **Supplementary Table S4**.

### Statistical Analysis

The incidence density and 95% CI of deaths in the study population based on the Poisson distribution were calculated. Risk factors for death after the COVID-19 suspicion date were evaluated with logistic regression (LR) models. A two-sided p-value <0.05 was considered statistically significant. The XGBoost (10) was also used to train and test a prediction model assessing the risk of death in the same cohort, an iterative method where, at each iteration, a new sub-model is added to correct the prediction error of the previous iteration. Each sub-model is an ensemble of decision trees. A decision tree can be roughly described as a flowchart-like structure in which each internal node represents a “discrimination test” on a given attribute (e.g., any clinical parameter or demographic characteristics); each branch of the decision tree represents the result of the discrimination test (i.e., passed/not passed), and each leaf node represents the probability of the outcome. This probability represents the prevalence of events occurring in each leaf in the training set. The iterative process ends in accordance with a pre-specified stopping rule (e.g., the maximum number of iterations or minimal acceptable average prediction error). The structure of the model is computed as a function optimization process combining the minimization of both training error and model complexity. XGBoost was selected since it is characterized by a good prediction accuracy in a broad variety of problems coupled with a short computational time. Furthermore, SHapley Additive exPlanations (SHAP) analysis enables intuitive model interpretation through an accurate and efficient estimation of the contribution of each input variable to the risk. To assess overfitting, the XGBoost model was trained in a dataset partition corresponding to 70% of records in the initial sample and tested in the remaining 30% of records. The discrimination of both the LR and the XGBoost model was assessed by calculating the area under the curve (AUC) of the receiver operating characteristics curve.

Both LR and XGBoost models were created for modifiable risk factors, as continuous parameters, and as indicator variables denoting target achievement according to KPIs as defined in **Supplementary Table S2**. In total, 4 models were created to assess whether the significance of modifiable factors was confirmed in both ML and LR models.

LR models were estimated using SAS software, Version 9.4 of the SAS System for Microsoft Windows. Copyright ^©^ 2013 SAS Institute Inc. SAS and all other SAS Institute Inc. products or service names are registered trademarks or trademarks of SAS Institute Inc., Cary, NC, USA. For XGBoost, the available open-source package for Python, Version 3.7.4 (Python Software Foundation, Delaware, DE, USA) was used.

## Results

### Sample Characteristics

**Figure 2:**
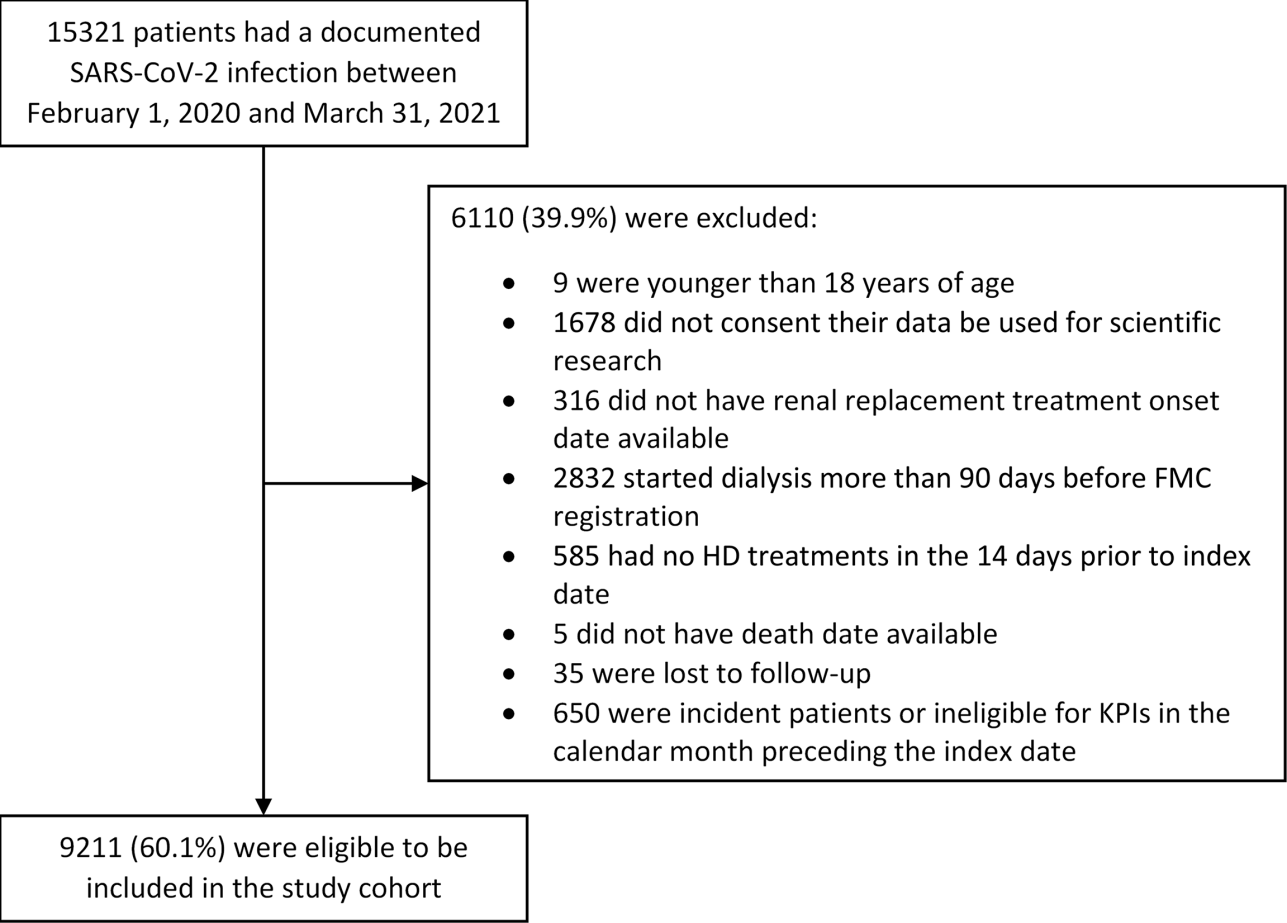
Study population and cohort enrollment process. The exclusion process was gradual: patients could have more than one reason for exclusion.

We observed 15,321 HD patients who had a documented SARS-CoV-2 infection between February 1, 2020, and March 31, 2021, in 588 dialysis centers located in 23 countries. Among these patients, 6,110 were excluded because they did not meet the inclusion criteria for the study. The exclusion process was gradual: patients could have more than one reason for exclusion. Nine were younger than 18 years at the index date, 16,78 did not consent for their data to be used for scientific research, 316 did not have renal replacement treatment onset date available, 2,832 started dialysis more than 90 days before FMC registration, 585 had no HD treatments in the 14 days prior to the index date, 5 did not have death date available, 35 were excluded because they were lost to follow-up, and 650 were incident patients or ineligible for KPIs in the calendar month preceding the index date. Therefore, 9,211 have been included in the study cohort coming from 552 dialysis centers located in 22 countries (**Figure 2**). The mean age was 65.4 ± 13.7 years, and the dialysis vintage was 4.2 ± 3.7 years. Patients’ characteristics at the index date are reported in **Table 1**.

**Table 1:** Characteristics of hemodialysis patients with COVID-19 disease at the time of suspicion date.

Characteristics	Whole sample
	(Mean ± SD, median) or n (%)
Patients	9,211
Age (years)	65.4 ± 13.7, 67.0
Sex (male)	58.7
Race	
Caucasian	6,008 (65.2)
Other	441 (4.8)
Missing	2,762 (30.0)
BMI (kg/m^2^)	28.7 ± 6.1, 27.9
Dialysis vintage (years)	4.2 ± 3.7, 3.2
Etiology:	
Diabetic nephropathy	1,691 (18.4)
Vascular disease hypertension	964 (10.5)
Glomerulonephritis	920 (10.0)
Chronic pyelonephritis	709 (7.7)
Cystic kidney disease	542 (5.9)
Miscellaneous	3,373 (36.6)
Missing	1,012 (11.0)
Comorbidities:	
Cardiovascular diseases	7,967 (86.5)
Genitourinary diseases	7,925 (86.0)
Diabetes	3,498 (38.0)
Digestive diseases	3,623 (39.3)
Infectious diseases	3,484 (37.8)
Respiratory diseases	2,827 (30.7)
Neoplasm and cancer	1,388 (15.1)
Gastrointestinal tract bleeding	491 (5.3)
COVID-19 vaccinated (at least 1 dose)	400 (4.34)
Smoking habit	
Current/past smoker	1,944 (21.1)
Non-smoker	4,284 (46.5)
Missing	2,983 (32.4)

Comorbidities were defined as the occurrence of suggestive ICD10 code prior to index date (**Supplementary Table S1**).

BMI, body mass index.

### Incidence of Death

We observed 1,912 deaths (20.8%, 95% CI: 19.9%–21.6%) within 30 days since the suspicion date among 9,211 patients with a confirmed diagnosis of COVID-19. The mortality rate was 238.2/1,000 person-months (95% CI: 227.7–249.1/1,000 person-months) over 8,027 months of follow-up overall.

### Non-Modifiable and Potentially Modifiable Factors Associated With Death

To assess the association between potentially modifiable factors during the ascertainment period and COVID-19-related death, we conducted an analysis by including KPIs as defined by the Medical Patient Review—Continuous Quality Improvement Program adopted in the FMC-NephroCare network. The LR model and the XGBoost model achieved comparable discrimination performance (LR: AUC = 0.72; XGBoost: AUC = 0.69). The LR showed that target achievement for serum albumin, serum sodium, erythropoietin resistance index, hydration status, hemodynamic status, Kt/V, inflammation, and serum phosphate were all associated with lower mortality risk (**Table 2**).

**Table 2:** Factors associated with 30-day COVID-19-related mortality including key performance indicators (KPIs) as defined by the Medical Patient Review—Continuous Quality Improvement Program adopted in the FMC-NephroCare network (**Supplementary Table S2**).

Characteristics	OR (95% CI)	p-Value	
***Non-modifiable factors***			
Age (years)	1.05 (1.04–1.05)	<0.0001	
Male	1.26 (1.11–1.43)	0.0004	
Race		0.1756	ns
Other vs Caucasian	1.27 (0.96–1.67)		
Missing vs Caucasian	0.97 (0.85–1.10)		
Dialysis vintage (years)	1.06 (1.04–1.08)	<0.0001	
Etiology		0.0575	ns
Vascular disease hypertension vs diabetic nephropathy	0.85 (0.68–1.06)		
Glomerulonephritis vs diabetic nephropathy	0.80 (0.62–1.03)		
Chronic pyelonephritis vs diabetic nephropathy	0.76 (0.59–0.99)		
Cystic kidney disease vs diabetic nephropathy	0.63 (0.46–0.87)		
Miscellaneous vs diabetic nephropathy	0.89 (0.75–1.07)		
Missing vs diabetic nephropathy	0.98 (0.79–1.21)		
Diabetes	1.31 (1.13–1.53)	0.0004	
Infectious diseases	1.15 (1.02–1.31)	0.0285	
Genitourinary diseases	1.23 (1.03–1.47)	0.0232	
Respiratory diseases	1.22 (1.07–1.39)	0.0025	
Neoplasm and cancer	0.88 (0.76–1.02)	0.0924	ns
***Potentially modifiable***			
BMI (kg/m^2^)		<0.0001	
>40 vs 19–25	1.85 (1.41–2.43)		
30–40 vs 19–25	1.28 (1.10–1.50)		
25–30 vs 19–25	0.98 (0.84–1.13)		
≤19 vs 19–25	1.21 (0.86–1.72)		
LTI (parameter off therapeutic target)	1.12 (1.00–1.26)	0.0483	
Smoking habit		0.0069	
Current/past smoker vs non-smoker	1.16 (1.00–1.34)		
Missing vs non-smoker	0.90 (0.79–1.03)		
Modality online HDF vs HD	0.86 (0.76–0.97)	0.0136	
Single-pool Kt/V (parameter off therapeutic target)	1.19 (1.02–1.38)	0.0246	
S-albumin (parameter off therapeutic target)	1.31 (1.11–1.54)	0.0012	
Hydration (parameter off therapeutic target)	1.15 (1.01–1.32)	0.0386	
Hemodynamic status (parameter off therapeutic target)	1.18 (1.04–1.35)	0.0132	
S-phosphate (mg/dl)		0.0077	
<2.5 vs [2.5, 5.5] (parameter off therapeutic target)	1.52 (1.07–2.16)		
≥5.5 vs [2.5, 5.5] (parameter off therapeutic target)	1.17 (1.01–1.35)		
ERI (parameter off therapeutic target)	1.28 (1.08–1.53)	0.0046	
C-reactive protein (parameter off therapeutic target)	1.20 (1.06–1.36)	0.0044	
S-sodium (parameter off therapeutic target)	1.12 (1.01–1.26)	0.0375	
WBC (Principal Component)	1.12 (1.06–1.19)	<0.0001	
Number of dialysis sessions (last 30 days)	0.95 (0.91–0.98)	0.0012	
Phosphate binders	0.86 (0.76–0.97)	0.0150	
Iron supplements	1.27 (1.00–1.61)	0.0496	
Cardiac vasodilators	1.24 (1.04–1.49)	0.0194	
Lipid-modifying agents	0.85 (0.75–0.97)	0.0154	
nPCR (parameter off therapeutic target)	1.11 (0.97–1.27)	0.1195	ns
S-bicarbonate (parameter off therapeutic target)	0.91 (0.81–1.03)	0.1523	ns

A p-value <0.05 was considered statistically significant; ns, p-value not significant at 0.05 level. WBC (Principal Component), white blood cell factor was calculated with a principal component analysis based on platelets, lymphocytes, and neutrophils, considering the first factor as explaining 47.2% of the total variance, basically a contrast of neutrophils (factor loading: 0.82865) against lymphocytes (−0.81652) with a very small loading on platelets (0.24885).

OR, odds ratio estimated by logistic regression; BMI, body mass index; LTI, lean tissue index; Online HDF, on-line hemodiafiltration; HD, conventional hemodialysis; ERI, erythropoietin resistance index; nPCR, normalized protein catabolic rate.

In the second model specification, we included biochemical markers as continuous variables. The LR model and the XGBoost model achieved comparable discrimination performance (LR: AUC = 0.71; XGBoost: AUC = 0.69). According to LR, non-modifiable factors associated with mortality risk were age, male sex, dialysis vintage and history of diabetes, infectious diseases, lung disease, and genitourinary diseases; among potentially modifiable factors, smoking habit, obesity, altered markers of inflammation, off target instead of inappropriate erythopoiesis-stimulating agents (ESA) administration, hyperphosphatemia, and overhydration were associated with increased mortality risk (**Table 3**).

**Table 3:** Factors associated with 30-day COVID-19-related mortality including biochemical markers as continuous variables.

Characteristics	OR (95% CI)	p-Value	
***Non-modifiable factors***			
Age (years)	1.05 (1.04–1.05)	<0.0001	
Male	1.34 (1.17–1.54)	<0.0001	
Race		0.0252	
Other vs Caucasian	1.41 (1.07–1.85)		
Missing vs Caucasian	0.96 (0.84–1.09)		
Dialysis vintage (years)	1.06 (1.05–1.08)	<0.0001	
Etiology		0.0291	
Vascular disease hypertension vs diabetic nephropathy	0.82 (0.66–1.03)		
Glomerulonephritis vs diabetic nephropathy	0.79 (0.62–1.02)		
Chronic pyelonephritis vs diabetic nephropathy	0.72 (0.55–0.93)		
Cystic kidney disease vs diabetic nephropathy	0.62 (0.45–0.84)		
Miscellaneous vs diabetic nephropathy	0.88 (0.74–1.05)		
Missing vs diabetic nephropathy	0.94 (0.76–1.17)		
Diabetes	1.32 (1.13–1.53)	0.0003	
Infectious diseases	1.15 (1.01–1.31)	0.0331	
Genitourinary diseases	1.22 (1.02–1.46)	0.0289	
Respiratory diseases	1.20 (1.06–1.37)	0.0050	
Neoplasm and cancer	0.86 (0.74–1.00)	0.0472	
***Potentially modifiable***			
BMI (kg/m^2^)	1.02 (1.01–1.03)	0.0061	
LTI (kg/m^2^)		0.3093	ns
<10% vs 10%–90% (parameter off therapeutic target)	1.15 (0.96–1.38)		
>90% vs 10%–90%	0.95 (0.76–1.18)		
FTI (kg/m^2^)		0.0260	
<10% vs 10%–90%	1.02 (0.81–1.27)		
>90% vs 10%–90%	1.33 (1.08–1.64)		
Smoking habit		0.0133	
Current/past smoker vs non-smoker	1.17 (1.01–1.35)		
Missing vs non-smoker	0.93 (0.81–1.06)		
Modality online HDF vs HD	0.89 (0.79–1.01)	0.0679	ns
Kt/V	0.83 (0.66–1.03)	0.0931	ns
S-Albumin (g/dl)	0.86 (0.73–1.00)	0.0556	ns
OH/ECW (%)	1.01 (1.00–1.01)	0.0471	
Pre-dialysis SBP (mmHg)	0.97 (0.94–1.00)*	0.0850	ns
S-phosphate (mg/dl)	1.05 (0.99–1.10)	0.1167	ns
ERI (parameter off therapeutic target)	1.36 (1.14–1.62)	0.0008	
C-reactive protein (mg/L)	1.00 (1.00–1.01)	0.0001	
S-sodium (mmol/L)	0.98 (0.96–1.00)	0.0823	ns
WBC (Principal Component)	1.12 (1.05–1.19)	0.0002	
Number of dialysis sessions (last 30 days)	0.94 (0.91–0.97)	0.0004	
Phosphate binders	0.85 (0.76–0.96)	0.0102	
Iron supplements	1.29 (1.01–1.63)	0.0392	
Cardiac vasodilators	1.27 (1.06–1.52)	0.0104	
Lipid-modifying agents	0.87 (0.77–0.99)	0.0361	
nPCR (g/kg/day)	0.71 (0.55–0.93)	0.0128	
S-bicarbonate (mmol/L)	0.98 (0.95–1.00)	0.0274	

A p-value <0.05 was considered statistically significant; ns, p-value not significant at 0.05 level. WBC (Principal Component), white blood cell factor was calculated with a principal component analysis based on platelets, lymphocytes, and neutrophils, considering the first factor as explaining 47.2% of the total variance, basically a contrast of neutrophils (factor loading: 0.82865) against lymphocytes (−0.81652) with a very small loading on platelets (0.24885).

OR, odds ratio estimated by logistic regression; BMI, body mass index; LTI, lean tissue index; FTI, fat tissue index; Online HDF, on-line hemodiafiltration; HD, conventional hemodialysis; OH/ECW, (relative overhydration)/extracellular water; SBP, systolic blood pressure: (*) this odds ratio is related to a 10 mmHg increase; ERI, erythropoietin resistance index; nPCR, normalized protein catabolic rate.

Whereas the primary analysis focusing on KPIs found that on-line hemodiafiltration was associated with lower mortality risk compared to conventional HD, the magnitude of this effect was similar but non-significant at the 0.05 level with the secondary model.

**Figure 3:**
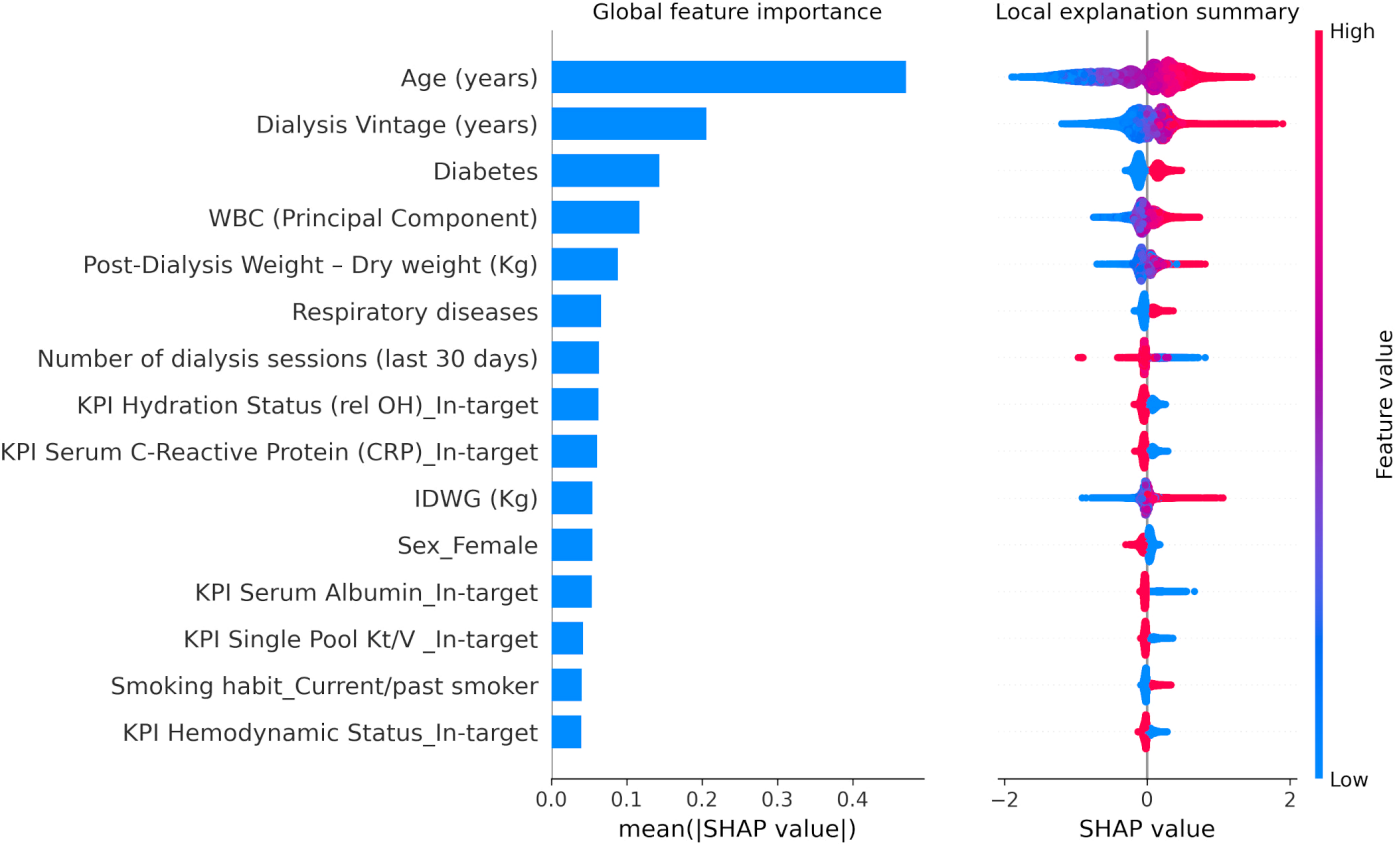
XGBoost SHapley Additive exPlanations (SHAP) related to 30-day COVID-19-related mortality including key performance indicators as defined by the Medical Patient Review—Continuous Quality Improvement Program adopted in the FMC-NephroCare network (**Supplementary Table S2**). WBC (Principal Component), white blood cell factor was calculated with a principal component analysis based on platelets, lymphocytes, and neutrophils, considering the first factor as explaining 47.2% of the total variance, basically a contrast of neutrophils (factor loading: 0.82865) against lymphocytes (−0.81652) with a very small loading on platelets (0.24885). IDWG, interdialytic weight gain. SHAP plots show relative feature importance for the 15 most important variables: the blue bar represents overall SHAP values and is interpreted as the relative importance of each variable to risk estimates. On the right side, SHAP values show the direction of association between predictor and risk estimates. Each dot represents one individual patient. Higher values of the predictors are represented in red color; lower values of the predictors are represented in blue color. The x-axis represents the impact of variables on risk in terms of SHAP values. Red color in correspondence with positive values suggests direct correlations between risk factors and 30-day COVID-19-related death, while red color in the region of negative SHAP values suggests inverse correlation.

**Figure 4:**
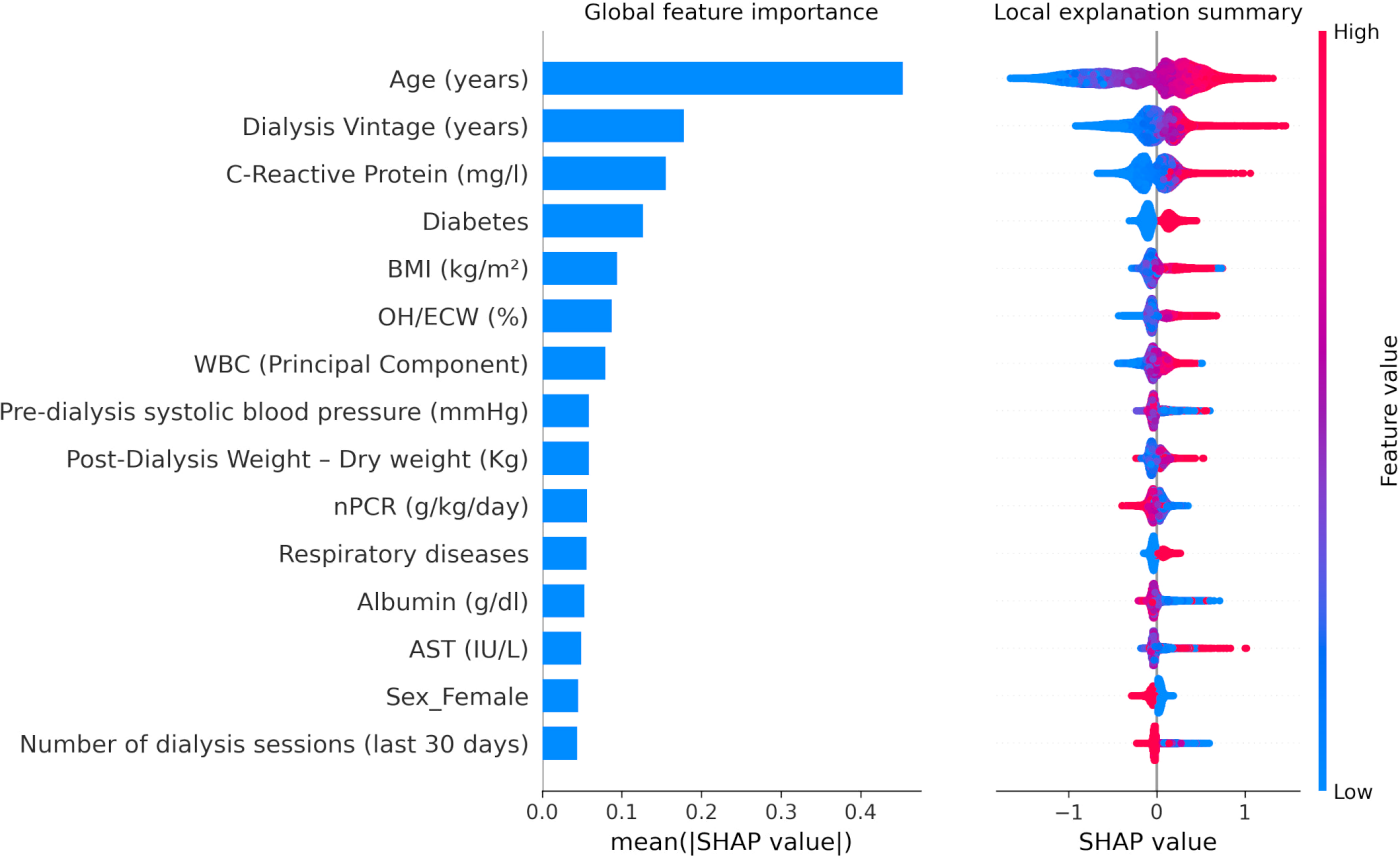
XGBoost SHapley Additive exPlanations (SHAP) related to 30-day COVID-19-related mortality including biochemical markers as continuous variables. BMI, body mass index; OH/ECW, overhydration normalized for extracellular water. WBC (Principal Component), white blood cell factor was calculated with a principal component analysis based on platelets, lymphocytes, and neutrophils, considering the first factor as explaining 47.2% of the total variance, basically a contrast of neutrophils (factor loading: 0.82865) against lymphocytes (−0.81652) with a very small loading on platelets (0.24885). nPCR, normalized protein catabolic rate. SHAP plots show relative feature importance for the 15 most important variables: the blue bar represents overall SHAP values and is interpreted as the relative importance of each variable to risk estimates. On the right side, SHAP values show the direction of association between predictor and risk estimates. Each dot represents one individual patient. Higher values of the predictors are represented in red color; lower values of the predictors are represented in blue color. The x-axis represents the impact of variables on risk in terms of SHAP values. Red color in correspondence with positive values suggests direct correlations between risk factors and 30-day COVID-19-related death, while red color in the region of negative SHAP values suggests inverse correlation.

A similar pattern of association with LR models was observed with XGBoost through SHAP value inspection (**Figures 3**, **Figure 4**). However, there were some notable differences in the relative importance of predictors between LR and XGBoost models. As an example, the presence of low serum phosphate levels was an important predictor of outcome in the LR model but did not emerge as a top 10 predictor in the XGBoost model.

In all models, the inclusion of COVID-19 vaccination status did not change parameter estimates for both modifiable and unmodifiable factors.

## Discussion

The present study is a large international cohort, applying both conventional statistical and ML models, confirming the importance of demographic factors, dialysis vintage, cause of renal failure, and comorbidities in association with 30-day COVID-related mortality. Even more importantly, the findings stressed the importance of potentially modifiable factors in COVID-19-related outcomes in patients with HD. In contrast to other studies, we did not collect these parameters at the onset of COVID-19 diagnosis but in the 6 months preceding the suspicion date, thus at a time when intervention theoretically might have been possible. The association with outcome was shown for risk factors in different domains and held true when potentially modifiable risk factors were analyzed as both KPIs and continuous parameters. We have previously shown that optimization of medical KPIs leads to improved survival among dialysis patients (11). In the present study, we provide evidence that this observation may hold true also during the COVID-19 pandemic.

Regarding potentially modifiable risk factors, all models showed that various parameters in the nutritional and inflammatory domains were important predictors of outcome. This was clearly expressed in the results of the XGBoost model, in which inflammatory and nutritional parameters had a strong contribution to the model. Markers of malnutrition, in which normalized protein catabolic rate (nPCR) and serum albumin were assessed during the ascertainment period, were inversely related to the study outcome. This is consistent with results from the general population in which malnutrition was also shown to be a major factor predicting the severity of COVID-19 (12). In contrast, both body mass index (BMI) above 30 kg/m^2^ and age-adjusted fat tissue index above the 90th percentile assessed by bioimpedance spectroscopy (BIS) were related to increased mortality risk. No association between lean tissue index and the outcome was observed. This contrasts with studies in HD patients without COVID-19 in which a low lean tissue index was related to increased mortality, especially in combination with a low fat tissue index (13). However, our study confirms the results of the ERACODA study, in which a BMI > 35 kg/m^2^ was associated with increased mortality (3). Thus, as also shown for the general population, obesity is also a risk factor for adverse outcomes of COVID-19 in patients with HD possibly associated with increased ACE2 receptors and disrupted adipokine expression, which mediates dysregulated inflammation in visceral fat adipocytes (14, 15).

A major risk factor for adverse outcomes was the presence of inflammation in the period before the COVID-19 suspicion date when expressed as both an increased neutrophil-to-lymphocyte ratio (e.g., white blood cell (WBC) factor in our analysis) and C-reactive protein, a finding consistent with previous studies of COVID-19-related mortality in the general population (16). Whereas the mechanism behind preexistent inflammation and COVID-19-related outcome cannot be deduced from the present study, it is tempting to speculate that immunosenescence and inflammaging, which are associated with chronic inflammation, might have played a role. It has been suggested that immunosenescence may both impair the initial defense against SARS-CoV-2 infection and play a role in the hyperinflammatory syndrome after COVID-19 (17).

Fluid overload, which is at least partially amenable to intervention, was also predictive of COVID-19-related outcomes. In our cohort, we had the availability of BIS measurements applied on a routine clinical basis. Fluid overload assessed by BIS is a consistent risk factor for outcomes in the general dialysis population (18). Whether the association with COVID-19 outcome is due to a higher risk for pulmonary complications due to an additive risk for pulmonary congestion (19) or to the association between fluid overload, malnutrition, and inflammation (20) cannot be assessed from the present study. Still, fluid overload turned out to be a major risk factor in the XGBoost model, with a major contribution to the model. Regarding other cardiovascular parameters, pre-dialysis systolic blood pressure (pre-HD SBP) was inversely related to mortality, albeit not significantly. The importance of low pre-HD SBP on short-term outcomes has been shown in previous studies in patients with HD and is likely for a major part related to underlying disease (21, 22). In contrast to the general population, high blood pressure was not related to increased mortality. Interestingly, the use of lipid-lowering agents was associated with reduced mortality. This agrees with a large observational study in the general population in Sweden (23), although the observed associations do not imply causation. Very important is the strong association between smoking and 30-day COVID-19-related mortality, which is also in agreement with studies on the general population.

Regarding parameters of mineral metabolism, our results showed that both increased and low serum phosphate levels were associated with increased mortality. We suggest that the effect of low serum phosphate levels (defined as levels between 2.5 mg/dl) on COVID-19-related outcomes might be explained by its association with malnutrition. We previously found that in combination with other markers of malnutrition, low serum phosphate levels were predictive of outcome in a large cohort of patients on HD (24).

Lastly, also dialysis prescription appears to be a relevant parameter related to outcome. This held true for single pool Kt/V when expressed as KPI, in which off-target values were related to increased mortality, as well as for on-line hemodiafiltration, which was protective in the model based on KPIs, although it did not reach significance in the model based on continuous parameters.

Our study differs from previous papers on this subject because we applied both conventional and ML models. The advantage of ML models such as XGBoost used in the present study is that they can, in contrast to more traditional models, handle both non-linear relationships and missing data. XGBoost is based on weak prediction trees, able to capture complex relationships in data without the need for high-order interactions or non-linear functions (25). Whereas with LR the association with the outcome is expressed by the odds ratio, in XGBoost, SHapley values are used for this purpose. SHAP values show the relative contribution of the different parameters to the model and thus yield additional information. In other studies, XGBoost outperformed conventional statistical techniques, e.g., in the prediction of volume responsiveness in patients with AKI (25). However, the discriminative value (expressed by AUC) of ML and LR models did not differ materially in our study, suggesting that no major cross-interaction or non-linear effects could improve prediction accuracy with the input variables considered. It may be argued that the AUC levels of the models were relatively similar, but it should be noted that the aim of the study was not to construct an optimal prediction model for COVID-19 outcomes but to assess the importance of factors in the period preceding COVID-19 suspicion date. Likely, stronger predictive models might be constructed using parameters at the time of the COVID-19 suspicion date, including disease-related factors. The differences in the relative importance of the individual parameters between the corresponding LR and ML models are explained by the intrinsic distinction in the two methodological techniques, both in the construction of the model and in the calculation of the importance of each variable.

Our study also adds to the literature because it is based on a large international cohort of dialysis providers, where parameters and KPIs are collected on a routine basis for quality assurance purposes. Previous studies based on large registries did not use detailed data on laboratory variables and body composition, as these are not usually collected. Drawbacks are the retrospective nature and the absence of data on frailty that were shown to be an important predictor of outcome in the ERACODA study (3). In addition, data were obtained mainly before the availability of vaccination; therefore, we were not able to evaluate the effect of potential cross-interaction between exposure to mRNA or viral carrier vaccine and KPI target achievement on COVID-19-related mortality risk. Whereas our results might have clinical implications in the sense that patients in whom KPIs were attained had a better outcome, this does not necessarily provide that targeted interventions in the time before SARS-CoV-2 infection might have improved outcome. However, in our opinion, our results stress the importance of adequate dialysis and supportive treatment also in the time of a pandemic.

In conclusion, this study showed, by using both conventional and ML models, the importance of modifiable risk factors, collected during the months preceding the COVID-19 suspicion date, in predicting COVID-19-related outcomes in patients on HD. Also, during the time of a pandemic, adequate dialysis and supportive treatment need to be maintained to the maximum extent possible.

## Data Availability Statement

The datasets presented in this article are not readily available because they include protected health information. Specific, well-motivated, requests to access the pseudo-anonymized analytic dataset may be considered by the authors. Requests to access the datasets should be directed to LN, Luca.Neri@fmc-ag.com.

## Ethics Statement

The studies involving human participants were reviewed and approved by Maastricht UMC METC azM/UM P. Debyelaan 25 Postbus 5800 6202 AZ Maastricht; Protocol number METC 2021-3077. The patients/participants provided their written informed consent for secondary analysis of their data.

## Author Contributions

Conceptualization: LN and JK. Data curation: PC. Formal analysis: PC. Supervision and validation: LN, JK, FB, and SS. Writing—original draft: JK, PC, and LN. Interpretation of results: all authors. Writing—review and editing: all authors. All authors have read and agreed to the published version of the manuscript.

## Conflict of Interest

PC, VK, OA, AW, YZ, FB, LU, JL, SS, and LN are employees of Fresenius Medical Care. PK and HZ are employees of the Renal Research Institute, a wholly owned subsidiary of Fresenius Medical Care. LU and PK have share options/ownership in Fresenius Medical Care. PK, HZ, LU, and JL are inventors on patents in the field of dialysis. JL is a guest editor on the Editorial Board of Frontiers in Physiology. PK receives an honorarium from HSTalks and is on the Editorial Boards of journals: Frontiers in Nephrology, Blood Purification, and Kidney and Blood Pressure Research.

The remaining author declares that the research was conducted in the absence of any commercial or financial relationships that could be construed as a potential conflict of interest.

## Publisher’s Note

All claims expressed in this article are solely those of the authors and do not necessarily represent those of their affiliated organizations, or those of the publisher, the editors and the reviewers. Any product that may be evaluated in this article, or claim that may be made by its manufacturer, is not guaranteed or endorsed by the publisher.
