# Supplementary material for: Modifiable Risk Factors Are Important Predictors of COVID-19-Related Mortality in Patients on Hemodialysis
Source: Front Nephrol. 2022 Jul 20;2:907959. doi: 10.3389/fneph.2022.907959 (PMC10479683; doi:10.3389/fneph.2022.907959)
Supplement: Supplementary file 1 [file Table_1.docx]

Supplementary Material

**Contents**

[1 Supplementary Table S1 – Definition of comorbidities 2](#_Toc97555868)

[2 Supplementary Table S2 – Definition of key performance healthcare indicators (KPIs) 9](#_Toc97555869)

[3 Supplementary Table S3 – Definition of medications 11](#_Toc97555870)

[4 Supplementary Table S4 – Data management 12](#_Toc97555871)

# Supplementary Table S1 – Definition of comorbidities

**Supplementary Table S1.** Comorbidities were defined as any occurrence prior to index date of suggestive ICD10 codes.

| **Diabetes** | |
| --- | --- |
|  | E10, E10.0, E10.1, E10.2, E10.3, E10.4, E10.5, E10.6, E10.7, E10.8, E10.9, E11, E11.0, E11.1, E11.2, E11.3, E11.4, E11.5, E11.6, E11.7, E11.8, E11.9, E12, E12.0, E12.1, E12.2, E12.3, E12.4, E12.5, E12.6, E12.7, E12.8, E12.9, E13, E13.0, E13.1, E13.2, E13.3, E13.4, E13.5, E13.6, E13.7, E13.8, E13.9, E14, E14.0, E14.1, E14.2, E14.3, E14.4, E14.5, E14.6, E14.7, E14.8, E14.9, O24, O24.0, O24.1, O24.2, O24.3, O24.4, O24.9, P70.2, R73, R73.0, R81 |
| **Infectious diseases** | |
|  | A00, A00.0, A00.1, A00.9, A01, A01.0, A01.1, A01.2, A01.3, A01.4, A02, A02.0, A02.1, A02.2, A02.8, A02.9, A03, A03.0, A03.1, A03.2, A03.3, A03.8, A03.9, A04, A04.0, A04.1, A04.2, A04.3, A04.4, A04.5, A04.6, A04.7, A04.8, A04.9, A05, A05.0, A05.1, A05.2, A05.3, A05.4, A05.8, A05.9, A06, A06.0, A06.1, A06.2, A06.3, A06.4, A06.5, A06.6, A06.7, A06.8, A06.9, A07, A07.0, A07.1, A07.2, A07.3, A07.8, A07.9, A08, A08.0, A08.1, A08.2, A08.3, A08.4, A08.5, A09, A09.0, A09.9, A15, A15.0, A15.1, A15.2, A15.3, A15.4, A15.5, A15.6, A15.7, A15.8, A15.9, A16, A16.0, A16.1, A16.2, A16.3, A16.4, A16.5, A16.7, A16.8, A16.9, A17, A17.0, A17.1, A17.8, A17.9, A18, A18.0, A18.1, A18.2, A18.3, A18.4, A18.5, A18.6, A18.7, A18.8, A19, A19.0, A19.1, A19.2, A19.8, A19.9, A20, A20.0, A20.1, A20.2, A20.3, A20.7, A20.8, A20.9, A21, A21.0, A21.1, A21.2, A21.3, A21.7, A21.8, A21.9, A22, A22.0, A22.1, A22.2, A22.7, A22.8, A22.9, A23, A23.0, A23.1, A23.2, A23.3, A23.8, A23.9, A24, A24.0, A24.1, A24.2, A24.3, A24.4, A25, A25.0, A25.1, A25.9, A26, A26.0, A26.7, A26.8, A26.9, A27, A27.0, A27.8, A27.9, A28, A28.0, A28.1, A28.2, A28.8, A28.9, A30, A30.0, A30.1, A30.2, A30.3, A30.4, A30.5, A30.8, A30.9, A31, A31.0, A31.1, A31.8, A31.9, A32, A32.0, A32.1, A32.7, A32.8, A32.9, A33, A34, A35, A36, A36.0, A36.1, A36.2, A36.3, A36.8, A36.9, A37, A37.0, A37.1, A37.8, A37.9, A38, A39, A39.0, A39.1, A39.2, A39.3, A39.4, A39.5, A39.8, A39.9, A40, A40.0, A40.1, A40.2, A40.3, A40.8, A40.9, A41, A41.0, A41.1, A41.2, A41.3, A41.4, A41.5, A41.8, A41.9, A42, A42.0, A42.1, A42.2, A42.7, A42.8, A42.9, A43, A43.0, A43.1, A43.8, A43.9, A44, A44.0, A44.1, A44.8, A44.9, A46, A48, A48.0, A48.1, A48.2, A48.3, A48.4, A48.8, A49, A49.0, A49.1, A49.2, A49.3, A49.8, A49.9, A50, A50.0, A50.1, A50.2, A50.3, A50.4, A50.5, A50.6, A50.7, A50.9, A51, A51.0, A51.1, A51.2, A51.3, A51.4, A51.5, A51.9, A52, A52.0, A52.1, A52.2, A52.3, A52.7, A52.8, A52.9, A53, A53.0, A53.9, A54, A54.0, A54.1, A54.2, A54.3, A54.4, A54.5, A54.6, A54.8, A54.9, A55, A56, A56.0, A56.1, A56.2, A56.3, A56.4, A56.8, A57, A58, A59, A59.0, A59.8, A59.9, A60, A60.0, A60.1, A60.9, A63, A63.0, A63.8, A64, A65, A66, A66.0, A66.1, A66.2, A66.3, A66.4, A66.5, A66.6, A66.7, A66.8, A66.9, A67, A67.0, A67.1, A67.2, A67.3, A67.9, A68, A68.0, A68.1, A68.9, A69, A69.0, A69.1, A69.2, A69.8, A69.9, A70, A71, A71.0, A71.1, A71.9, A74, A74.0, A74.8, A74.9, A75, A75.0, A75.1, A75.2, A75.3, A75.9, A77, A77.0, A77.1, A77.2, A77.3, A77.8, A77.9, A78, A79, A79.0, A79.1, A79.8, A79.9, A80, A80.0, A80.1, A80.2, A80.3, A80.4, A80.9, A81, A81.0, A81.1, A81.2, A81.8, A81.9, A82, A82.0, A82.1, A82.9, A83, A83.0, A83.1, A83.2, A83.3, A83.4, A83.5, A83.6, A83.8, A83.9, A84, A84.0, A84.1, A84.8, A84.9, A85, A85.0, A85.1, A85.2, A85.8, A86, A87, A87.0, A87.1, A87.2, A87.8, A87.9, A88, A88.0, A88.1, A88.8, A89, A92, A92.0, A92.1, A92.2, A92.3, A92.4, A92.5, A92.8, A92.9, A93, A93.0, A93.1, A93.2, A93.8, A94, A95, A95.0, A95.1, A95.9, A96, A96.0, A96.1, A96.2, A96.8, A96.9, A97, A97.0, A97.1, A97.2, A97.9, A98, A98.0, A98.1, A98.2, A98.3, A98.4, A98.5, A98.8, A99, B00, B00.0, B00.1, B00.2, B00.3, B00.4, B00.5, B00.7, B00.8, B00.9, B01, B01.0, B01.1, B01.2, B01.8, B01.9, B02, B02.0, B02.1, B02.2, B02.3, B02.7, B02.8, B02.9, B03, B04, B05, B05.0, B05.1, B05.2, B05.3, B05.4, B05.8, B05.9, B06, B06.0, B06.8, B06.9, B07, B08, B08.0, B08.1, B08.2, B08.3, B08.4, B08.5, B08.8, B09, B15, B15.0, B15.9, B16, B16.0, B16.1, B16.2, B16.9, B17, B17.0, B17.1, B17.2, B17.8, B17.9, B18, B18.0, B18.00, B18.09, B18.1, B18.10, B18.19, B18.2, B18.8, B18.9, B19, B19.0, B19.9, B20, B20.0, B20.1, B20.2, B20.3, B20.4, B20.5, B20.6, B20.7, B20.8, B20.9, B21, B21.0, B21.1, B21.2, B21.3, B21.7, B21.8, B21.9, B22, B22.0, B22.1, B22.2, B22.7, B23, B23.0, B23.1, B23.2, B23.8, B24, B25, B25.0, B25.1, B25.2, B25.8, B25.9, B26, B26.0, B26.1, B26.2, B26.3, B26.8, B26.9, B27, B27.0, B27.1, B27.8, B27.9, B30, B30.0, B30.1, B30.2, B30.3, B30.8, B30.9, B33, B33.0, B33.1, B33.2, B33.3, B33.4, B33.8, B34, B34.0, B34.1, B34.2, B34.3, B34.4, B34.8, B34.9, B35, B35.0, B35.1, B35.2, B35.3, B35.4, B35.5, B35.6, B35.8, B35.9, B36, B36.0, B36.1, B36.2, B36.3, B36.8, B36.9, B37, B37.0, B37.1, B37.2, B37.3, B37.4, B37.5, B37.6, B37.7, B37.8, B37.9, B38, B38.0, B38.1, B38.2, B38.3, B38.4, B38.7, B38.8, B38.9, B39, B39.0, B39.1, B39.2, B39.3, B39.4, B39.5, B39.9, B40, B40.0, B40.1, B40.2, B40.3, B40.7, B40.8, B40.9, B41, B41.0, B41.7, B41.8, B41.9, B42, B42.0, B42.1, B42.7, B42.8, B42.9, B43, B43.0, B43.1, B43.2, B43.8, B43.9, B44, B44.0, B44.1, B44.2, B44.7, B44.8, B44.9, B45, B45.0, B45.1, B45.2, B45.3, B45.7, B45.8, B45.9, B46, B46.0, B46.1, B46.2, B46.3, B46.4, B46.5, B46.8, B46.9, B47, B47.0, B47.1, B47.9, B48, B48.0, B48.1, B48.2, B48.3, B48.4, B48.5, B48.7, B48.8, B49, B50, B50.0, B50.8, B50.9, B51, B51.0, B51.8, B51.9, B52, B52.0, B52.8, B52.9, B53, B53.0, B53.1, B53.8, B54, B55, B55.0, B55.1, B55.2, B55.9, B56, B56.0, B56.1, B56.9, B57, B57.0, B57.1, B57.2, B57.3, B57.4, B57.5, B58, B58.0, B58.1, B58.2, B58.3, B58.8, B58.9, B60, B60.0, B60.1, B60.2, B60.8, B64, B65, B65.0, B65.1, B65.2, B65.3, B65.8, B65.9, B66, B66.0, B66.1, B66.2, B66.3, B66.4, B66.5, B66.8, B66.9, B67, B67.0, B67.1, B67.2, B67.3, B67.4, B67.5, B67.6, B67.7, B67.8, B67.9, B68, B68.0, B68.1, B68.9, B69, B69.0, B69.1, B69.8, B69.9, B70, B70.0, B70.1, B71, B71.0, B71.1, B71.8, B71.9, B72, B73, B74, B74.0, B74.1, B74.2, B74.3, B74.4, B74.8, B74.9, B75, B76, B76.0, B76.1, B76.8, B76.9, B77, B77.0, B77.8, B77.9, B78, B78.0, B78.1, B78.7, B78.9, B79, B80, B81, B81.0, B81.1, B81.2, B81.3, B81.4, B81.8, B82, B82.0, B82.9, B83, B83.0, B83.1, B83.2, B83.3, B83.4, B83.8, B83.9, B85, B85.0, B85.1, B85.2, B85.3, B85.4, B86, B87, B87.0, B87.1, B87.2, B87.3, B87.4, B87.8, B87.9, B88, B88.0, B88.1, B88.2, B88.3, B88.8, B88.9, B89, B90, B90.0, B90.1, B90.2, B90.8, B90.9, B91, B92, B94, B94.0, B94.1, B94.2, B94.8, B94.9, B95, B95.0, B95.1, B95.2, B95.3, B95.4, B95.5, B95.6, B95.7, B95.8, B96, B96.0, B96.1, B96.2, B96.3, B96.4, B96.5, B96.6, B96.7, B96.8, B97, B97.0, B97.1, B97.2, B97.3, B97.4, B97.5, B97.6, B97.7, B97.8, B98, B98.0, B98.1, B99, C02.0, D86, D86.0, D86.1, D86.2, D86.3, D86.8, D86.9, E32.1, F02.8, G00, G00.0, G00.1, G00.2, G00.3, G00.8, G00.9, G01, G02, G02.0, G02.1, G02.8, G03, G03.0, G03.1, G03.8, G03.9, G04, G04.0, G04.2, G04.8, G04.9, G05, G05.0, G05.1, G05.2, G05.8, G06, G06.0, G06.1, G06.2, G07, G08, G09, G14, G37.4, G92, H00, H00.0, H00.1, H01.0, H02.3, H10, H10.0, H10.1, H10.2, H10.3, H10.4, H10.5, H10.8, H10.9, H32.0, H60.0, H60.1, H60.2, H60.3, H66, H66.0, H66.1, H66.2, H66.3, H66.4, H67.0, H67.1, H67.8, H70.0, H70.1, H70.2, H75.0, H83.0, I00, I01, I01.0, I01.1, I01.2, I01.8, I01.9, I02, I02.0, I02.9, I05, I06, I06.0, I06.1, I06.2, I06.8, I09.0, I09.1, I09.2, I09.8, I09.9, I30.1, I32, I32.0, I32.1, I32.8, I33.0, I40.0, I41, J00, J01, J01.0, J01.1, J01.2, J01.3, J01.4, J01.8, J01.9, J02, J02.0, J02.8, J02.9, J03, J03.0, J03.8, J03.9, J04, J04.0, J04.1, J04.2, J05, J05.0, J05.1, J06, J06.0, J06.8, J06.9, J09, J10, J10.0, J10.1, J10.8, J11, J11.0, J11.1, J11.8, J12, J12.0, J12.1, J12.2, J12.3, J12.8, J12.9, J13, J14, J15, J15.0, J15.1, J15.2, J15.3, J15.4, J15.5, J15.6, J15.7, J15.8, J15.9, J16, J16.0, J16.8, J17, J17.0, J17.1, J17.2, J17.3, J17.8, J18, J18.0, J18.1, J18.2, J18.8, J18.9, J20, J20.0, J20.1, J20.2, J20.3, J20.4, J20.5, J20.6, J20.7, J20.8, J20.9, J21, J21.0, J21.1, J21.8, J21.9, J22, J31, J31.0, J31.1, J31.2, J32, J32.0, J32.1, J32.2, J32.3, J32.4, J32.8, J34.0, J35, J35.0, J36, J37, J37.0, J37.1, J39.0, J39.1, J40, J41, J41.0, J41.1, J41.8, J42, J85, J85.0, J85.1, J85.2, J85.3, J86, J86.0, J86.9, K04.6, K04.7, K11.3, K12.2, K35.2, K35.3, K35.8, K36, K37, K50.0, K50.1, K50.9, K51.0, K51.2, K51.3, K51.4, K51.8, K51.9, K57.0, K57.1, K57.2, K57.3, K57.4, K57.5, K57.8, K57.9, K61, K61.0, K61.1, K61.2, K61.3, K61.4, K63.0, K65, K65.0, K65.8, K67, K67.0, K67.1, K67.2, K67.3, K67.8, K75.0, K75.1, K81.0, K81.1, K81.9, L00, L01, L02, L02.0, L02.1, L02.2, L02.3, L02.4, L02.8, L02.9, L03, L03.0, L03.1, L03.2, L03.3, L03.8, L03.9, L04, L04.0, L04.1, L04.2, L04.3, L04.8, L04.9, L05, L05.0, L05.9, L08, L08.0, L08.1, L08.8, L08.9, L44.4, L70.2, L88, L92.8, L94.6, L98.0, L98.3, M00, M00.0, M00.1, M00.2, M00.8, M00.9, M01, M01.0, M01.1, M01.2, M01.3, M01.4, M01.5, M01.6, M01.8, M02.1, M02.3, M35.2, M46.2, M46.3, M60.0, M71.0, M86.0, M86.1, M86.2, M86.3, M86.4, M86.5, M86.6, M86.8, M86.9, M90.0, M90.1, M90.2, N10, N11.0, N11.1, N12, N13.6, N15.1, N30, N30.0, N34.0, N34.1, N34.2, N34.3, N35.1, N37, N41.0, N41.1, N41.2, N41.3, N45, N45.0, N45.9, N48.1, N49.0, N49.1, N49.2, N49.8, N61, N70, N70.0, N71.0, N71.1, N72, N73.0, N73.1, N73.2, N73.3, N73.4, N73.5, N74, N75.1, N76.0, N76.1, N76.2, N76.3, N76.4, N77.1, N98.0, O03.0, O03.5, O04.5, O05.0, O05.5, O06.0, O06.5, O07.0, O07.5, O08.0, O23, O23.0, O23.1, O23.2, O23.3, O23.4, O23.5, O23.9, O41.1, O75.3, O86, O86.0, O86.1, O86.2, O86.3, O86.8, O91, O91.0, O91.1, O98, O98.0, O98.1, O98.2, O98.3, O98.4, O98.5, O98.6, O98.7, O98.8, O98.9, P23, P23.0, P23.1, P23.2, P23.3, P23.4, P23.5, P23.6, P23.8, P23.9, P35, P35.0, P35.1, P35.2, P35.3, P35.4, P35.8, P35.9, P36, P36.0, P36.1, P36.2, P36.3, P36.4, P36.5, P36.8, P36.9, P37, P37.0, P37.1, P37.2, P37.3, P37.4, P37.5, P37.8, P37.9, P38, P39, P39.0, P39.1, P39.2, P39.3, P39.4, P39.8, P39.9, P58.2, R75, T79.3, T80.2, T81.4, T82.6, T82.7, T83.5, T83.6, T84.5, T84.6, T84.7, T85.7, T87.4, T88.0, Z11.4, Z20.2, Z20.6, Z21, Z71.7, Z83.0 |
| **Cardiovascular diseases** | |
|  | E10.5, E11.5, E12.5, E13.5, E14.5, G45, G45.0, G45.1, G45.2, G45.3, G45.4, G45.8, G45.9, G46, G46.0, G46.1, G46.2, G46.3, G46.4, G46.5, G46.6, G46.7, G46.8, H34, H34.0, H34.1, H34.2, H34.8, H34.9, I00, I01, I01.0, I01.1, I01.2, I01.8, I01.9, I02.0, I05, I05.0, I05.1, I05.2, I05.8, I05.9, I06, I06.0, I06.1, I06.2, I06.8, I06.9, I07, I07.0, I07.1, I07.2, I07.8, I07.9, I08, I08.0, I08.1, I08.2, I08.3, I08.8, I08.9, I09, I09.0, I09.1, I09.2, I09.8, I09.9, I10, I11, I11.0, I11.9, I12, I12.0, I12.9, I13, I13.0, I13.1, I13.2, I13.9, I15, I15.0, I15.1, I15.2, I15.8, I15.9, I20, I20.0, I20.1, I20.8, I20.9, I21, I21.0, I21.1, I21.2, I21.3, I21.4, I21.9, I22, I22.0, I22.1, I22.8, I22.9, I23, I23.0, I23.1, I23.2, I23.3, I23.4, I23.5, I23.6, I23.8, I24, I24.0, I24.1, I24.8, I24.9, I25, I25.0, I25.1, I25.2, I25.3, I25.4, I25.5, I25.6, I25.8, I25.9, I26, I26.0, I26.9, I27, I27.0, I27.1, I27.2, I27.8, I27.9, I28, I28.0, I28.1, I28.8, I28.9, I30, I30.0, I30.1, I30.8, I30.9, I31, I31.0, I31.1, I31.2, I31.3, I31.8, I31.9, I32, I32.0, I32.1, I32.8, I33, I33.0, I33.9, I34, I34.0, I34.1, I34.2, I34.8, I34.9, I35, I35.0, I35.1, I35.2, I35.8, I35.9, I36, I36.0, I36.1, I36.2, I36.8, I36.9, I37, I37.0, I37.1, I37.2, I37.8, I37.9, I38, I39, I39.0, I39.1, I39.2, I39.3, I39.4, I39.8, I40, I40.0, I40.1, I40.8, I40.9, I41, I41.0, I41.1, I41.2, I41.8, I42, I42.0, I42.1, I42.2, I42.3, I42.4, I42.5, I42.6, I42.7, I42.8, I42.9, I43, I43.0, I43.1, I43.2, I43.8, I44, I44.0, I44.1, I44.2, I44.3, I44.4, I44.5, I44.6, I44.7, I45, I45.0, I45.1, I45.2, I45.3, I45.4, I45.5, I45.6, I45.8, I45.9, I46, I46.0, I46.1, I46.9, I47, I47.0, I47.1, I47.2, I47.9, I48, I48.0, I48.1, I48.2, I48.3, I48.4, I48.9, I49, I49.0, I49.1, I49.2, I49.3, I49.4, I49.5, I49.8, I49.9, I50, I50.0, I50.1, I50.9, I51, I51.0, I51.1, I51.2, I51.3, I51.4, I51.5, I51.6, I51.7, I51.8, I51.9, I52, I52.0, I52.1, I52.8, I60, I60.0, I60.1, I60.2, I60.3, I60.4, I60.5, I60.6, I60.7, I60.8, I60.9, I61, I61.0, I61.1, I61.2, I61.3, I61.4, I61.5, I61.6, I61.8, I61.9, I62, I62.0, I62.1, I62.9, I63, I63.0, I63.1, I63.2, I63.3, I63.4, I63.5, I63.6, I63.8, I63.9, I64, I65, I65.0, I65.1, I65.2, I65.3, I65.8, I65.9, I66, I66.0, I66.1, I66.2, I66.3, I66.4, I66.8, I66.9, I67, I67.0, I67.1, I67.2, I67.3, I67.4, I67.5, I67.6, I67.7, I67.8, I67.9, I68, I68.0, I68.1, I68.2, I68.8, I69, I69.0, I69.1, I69.2, I69.3, I69.4, I69.8, I70, I70.0, I70.1, I70.2, I70.8, I70.9, I71, I71.0, I71.1, I71.2, I71.3, I71.4, I71.5, I71.6, I71.8, I71.9, I72, I72.0, I72.1, I72.2, I72.3, I72.4, I72.5, I72.6, I72.8, I72.9, I73, I73.0, I73.1, I73.8, I73.9, I74, I74.0, I74.1, I74.2, I74.3, I74.4, I74.5, I74.8, I74.9, I77, I77.0, I77.1, I77.2, I77.3, I77.4, I77.5, I77.6, I77.8, I77.9, I78, I78.0, I78.1, I78.8, I78.9, I79, I79.0, I79.1, I79.2, I79.8, I80, I80.0, I80.1, I80.2, I80.3, I80.8, I80.9, I81, I82, I82.0, I82.1, I82.2, I82.3, I82.8, I82.9, I83, I83.0, I83.1, I83.2, I83.9, I85, I85.0, I85.9, I86, I86.0, I86.1, I86.2, I86.3, I86.4, I86.8, I87, I87.0, I87.1, I87.2, I87.8, I87.9, I88, I88.0, I88.1, I88.8, I88.9, I89, I89.0, I89.1, I89.8, I89.9, I95, I95.0, I95.1, I95.2, I95.8, I95.9, I97, I97.0, I97.1, I97.2, I97.8, I97.9, I98, I98.0, I98.1, I98.2, I98.3, I98.8, I99, K76.1, K76.2, M31.8, M31.9, O22.5, O22.8, O22.9, O87.3, O87.8, O87.9, O90.3, O99.4, P29.0, R00.0, R00.1, R00.2, T81.7 |
| **Digestive diseases** | |
|  | K00, K00.0, K00.1, K00.2, K00.3, K00.4, K00.5, K00.6, K00.7, K00.8, K00.9, K01, K01.0, K01.1, K02, K02.0, K02.1, K02.2, K02.3, K02.4, K02.5, K02.8, K02.9, K03, K03.0, K03.1, K03.2, K03.3, K03.4, K03.5, K03.6, K03.7, K03.8, K03.9, K04, K04.0, K04.1, K04.2, K04.3, K04.4, K04.5, K04.6, K04.7, K04.8, K04.9, K05, K05.0, K05.1, K05.2, K05.3, K05.4, K05.5, K05.6, K06, K06.0, K06.1, K06.2, K06.8, K06.9, K07, K07.0, K07.1, K07.2, K07.3, K07.4, K07.5, K07.6, K07.8, K07.9, K08, K08.0, K08.1, K08.2, K08.3, K08.8, K08.9, K09, K09.0, K09.1, K09.2, K09.8, K09.9, K10, K10.0, K10.1, K10.2, K10.3, K10.8, K10.9, K11, K11.0, K11.1, K11.2, K11.3, K11.4, K11.5, K11.6, K11.7, K11.8, K11.9, K12, K12.0, K12.1, K12.2, K12.3, K13, K13.0, K13.1, K13.2, K13.3, K13.4, K13.5, K13.6, K13.7, K14, K14.0, K14.1, K14.2, K14.3, K14.4, K14.5, K14.6, K14.8, K14.9, K20, K21, K21.0, K21.9, K22, K22.0, K22.1, K22.2, K22.3, K22.4, K22.5, K22.6, K22.7, K22.8, K22.9, K23, K23.0, K23.1, K23.8, K25, K25.0, K25.1, K25.2, K25.3, K25.4, K25.5, K25.6, K25.7, K25.9, K26, K26.0, K26.1, K26.2, K26.3, K26.4, K26.5, K26.6, K26.7, K26.9, K27, K27.0, K27.1, K27.2, K27.3, K27.4, K27.5, K27.6, K27.7, K27.9, K28, K28.0, K28.1, K28.2, K28.3, K28.4, K28.5, K28.6, K28.7, K28.9, K29, K29.0, K29.1, K29.2, K29.3, K29.4, K29.5, K29.6, K29.7, K29.8, K29.9, K30, K31, K31.0, K31.1, K31.2, K31.3, K31.4, K31.5, K31.6, K31.7, K31.8, K31.9, K35, K35.2, K35.3, K35.8, K36, K37, K38, K38.0, K38.1, K38.2, K38.3, K38.8, K38.9, K40, K40.0, K40.1, K40.2, K40.3, K40.4, K40.9, K41, K41.0, K41.1, K41.2, K41.3, K41.4, K41.9, K42, K42.0, K42.1, K42.9, K43, K43.0, K43.1, K43.2, K43.3, K43.4, K43.5, K43.6, K43.7, K43.9, K44, K44.0, K44.1, K44.9, K45, K45.0, K45.1, K45.8, K46, K46.0, K46.1, K46.9, K50, K50.0, K50.1, K50.8, K50.9, K51, K51.0, K51.2, K51.3, K51.4, K51.5, K51.8, K51.9, K52, K52.0, K52.1, K52.2, K52.3, K52.8, K52.9, K55, K55.0, K55.1, K55.2, K55.3, K55.8, K55.9, K56, K56.0, K56.1, K56.2, K56.3, K56.4, K56.5, K56.6, K56.7, K57, K57.0, K57.1, K57.2, K57.3, K57.4, K57.5, K57.8, K57.9, K58, K58.1, K58.2, K58.3, K58.8, K59, K59.0, K59.1, K59.2, K59.3, K59.4, K59.8, K59.9, K60, K60.0, K60.1, K60.2, K60.3, K60.4, K60.5, K61, K61.0, K61.1, K61.2, K61.3, K61.4, K62, K62.0, K62.1, K62.2, K62.3, K62.4, K62.5, K62.6, K62.7, K62.8, K62.9, K63, K63.0, K63.1, K63.2, K63.3, K63.4, K63.5, K63.8, K63.9, K64, K64.0, K64.1, K64.2, K64.3, K64.4, K64.5, K64.8, K64.9, K65, K65.0, K65.8, K65.9, K66, K66.0, K66.1, K66.2, K66.8, K66.9, K67, K67.0, K67.1, K67.2, K67.3, K67.8, K70, K70.0, K70.1, K70.2, K70.3, K70.4, K70.9, K71, K71.0, K71.1, K71.2, K71.3, K71.4, K71.5, K71.6, K71.7, K71.8, K71.9, K72, K72.0, K72.1, K72.9, K73, K73.0, K73.1, K73.2, K73.8, K73.9, K74, K74.0, K74.1, K74.2, K74.3, K74.4, K74.5, K74.6, K75, K75.0, K75.1, K75.2, K75.3, K75.4, K75.8, K75.9, K76, K76.0, K76.1, K76.2, K76.3, K76.4, K76.5, K76.6, K76.7, K76.8, K76.9, K77, K77.0, K77.8, K80, K80.0, K80.1, K80.2, K80.3, K80.4, K80.5, K80.8, K81, K81.0, K81.1, K81.8, K81.9, K82, K82.0, K82.1, K82.2, K82.3, K82.4, K82.8, K82.9, K83, K83.0, K83.1, K83.2, K83.3, K83.4, K83.5, K83.8, K83.9, K85, K85.0, K85.1, K85.2, K85.3, K85.8, K85.9, K86, K86.0, K86.1, K86.2, K86.3, K86.8, K86.9, K87, K87.0, K87.1, K90, K90.0, K90.1, K90.2, K90.3, K90.4, K90.8, K90.9, K91, K91.0, K91.1, K91.2, K91.3, K91.4, K91.5, K91.8, K91.9, K92, K92.0, K92.1, K92.2, K92.8, K92.9, K93, K93.0, K93.1, K93.8, P75, P76, P76.0, P76.1, P76.2, P76.8, P76.9, P77, P78, Q38, Q38.0, Q38.1, Q38.2, Q38.3, Q38.4, Q38.5, Q38.6, Q38.7, Q38.8, Q39, Q39.0, Q39.1, Q39.2, Q39.3, Q39.4, Q39.5, Q39.6, Q39.8, Q39.9, Q40, Q40.0, Q40.1, Q40.2, Q40.3, Q40.8, Q40.9, Q41, Q41.0, Q41.1, Q41.2, Q41.8, Q41.9, Q42, Q42.0, Q42.1, Q42.2, Q42.3, Q42.8, Q42.9, Q43, Q43.0, Q43.1, Q43.2, Q43.3, Q43.4, Q43.5, R10, R10.0, R10.1, R10.2, R10.3, R10.4, R11, R12, R13, R14, R15, R16, R16.0, R16.1, R16.2, R17, R17.0, R17.9, R18, R19, R19.0, R19.1, R19.2, R19.3, R19.4, R19.5, R19.6, R19.8 |
| **Genitourinary diseases** | |
|  | N00, N00.0, N00.1, N00.2, N00.3, N00.4, N00.5, N00.6, N00.7, N00.8, N00.9, N01, N01.0, N01.1, N01.2, N01.3, N01.4, N01.5, N01.6, N01.7, N01.8, N01.9, N02, N02.0, N02.1, N02.2, N02.3, N02.4, N02.5, N02.6, N02.7, N02.8, N02.9, N03, N03.0, N03.1, N03.2, N03.3, N03.4, N03.5, N03.6, N03.7, N03.8, N03.9, N04, N04.0, N04.1, N04.2, N04.3, N04.4, N04.5, N04.6, N04.7, N04.8, N04.9, N05, N05.0, N05.1, N05.2, N05.3, N05.4, N05.5, N05.6, N05.7, N05.8, N05.9, N06, N06.0, N06.1, N06.2, N06.3, N06.4, N06.5, N06.6, N06.7, N06.8, N06.9, N07, N07.0, N07.1, N07.2, N07.3, N07.4, N07.5, N07.6, N07.7, N07.8, N07.9, N08, N08.0, N08.1, N08.2, N08.3, N08.4, N08.5, N08.8, N10, N11, N11.0, N11.1, N11.8, N11.9, N12, N13, N13.0, N13.1, N13.2, N13.3, N13.4, N13.5, N13.6, N13.7, N13.8, N13.9, N14, N14.0, N14.1, N14.2, N14.3, N14.4, N15, N15.0, N15.1, N15.8, N15.9, N16, N16.0, N16.1, N16.2, N16.3, N16.4, N16.5, N16.8, N17, N17.0, N17.1, N17.2, N17.8, N17.9, N18, N18.1, N18.2, N18.3, N18.4, N18.5, N18.9, N19, N20, N20.0, N20.1, N20.2, N20.9, N21, N21.0, N21.1, N21.8, N21.9, N22, N22.0, N22.8, N23, N25, N25.0, N25.1, N25.8, N25.9, N26, N27, N27.0, N27.1, N27.9, N28, N28.0, N28.1, N28.8, N28.9, N29, N29.0, N29.1, N29.8, N30, N30.0, N30.1, N30.2, N30.3, N30.4, N30.8, N30.9, N31, N31.0, N31.1, N31.2, N31.8, N31.9, N32, N32.0, N32.1, N32.2, N32.3, N32.4, N32.8, N32.9, N33, N33.0, N33.8, N34, N34.0, N34.1, N34.2, N34.3, N35, N35.0, N35.1, N35.8, N35.9, N36, N36.0, N36.1, N36.2, N36.3, N36.8, N36.9, N37, N37.0, N37.8, N39, N39.0, N39.1, N39.2, N39.3, N39.4, N39.8, N39.9, N40, N41, N41.0, N41.1, N41.2, N41.3, N41.8, N41.9, N42, N42.0, N42.1, N42.2, N42.3, N42.8, N42.9, N43, N43.0, N43.1, N43.2, N43.3, N43.4, N44, N45, N45.0, N45.9, N46, N47, N48, N48.0, N48.1, N48.2, N48.3, N48.4, N48.5, N48.6, N48.8, N48.9, N49, N49.0, N49.1, N49.2, N49.8, N49.9, N50, N50.0, N50.1, N50.8, N50.9, N51, N51.0, N51.1, N51.2, N51.8, N60, N60.0, N60.1, N60.2, N60.3, N60.4, N60.8, N60.9, N61, N62, N63, N64, N64.0, N64.1, N64.2, N64.3, N64.4, N64.5, N64.8, N64.9, N70, N70.0, N70.1, N70.9, N71, N71.0, N71.1, N71.9, N72, N73, N73.0, N73.1, N73.2, N73.3, N73.4, N73.5, N73.6, N73.8, N73.9, N74, N74.0, N74.1, N74.2, N74.3, N74.4, N74.8, N75, N75.0, N75.1, N75.8, N75.9, N76, N76.0, N76.1, N76.2, N76.3, N76.4, N76.5, N76.6, N76.8, N77, N77.0, N77.1, N77.8, N80, N80.0, N80.1, N80.2, N80.3, N80.4, N80.5, N80.6, N80.8, N80.9, N81, N81.0, N81.1, N81.2, N81.3, N81.4, N81.5, N81.6, N81.8, N81.9, N82, N82.0, N82.1, N82.2, N82.3, N82.4, N82.5, N82.8, N82.9, N83, N83.0, N83.1, N83.2, N83.3, N83.4, N83.5, N83.6, N83.7, N83.8, N83.9, N84, N84.0, N84.1, N84.2, N84.3, N84.8, N84.9, N85, N85.0, N85.1, N85.2, N85.3, N85.4, N85.5, N85.6, N85.7, N85.8, N85.9, N86, N87, N87.0, N87.1, N87.2, N87.9, N88, N88.0, N88.1, N88.2, N88.3, N88.4, N88.8, N88.9, N89, N89.0, N89.1, N89.2, N89.3, N89.4, N89.5, N89.6, N89.7, N89.8, N89.9, N90, N90.0, N90.1, N90.2, N90.3, N90.4, N90.5, N90.6, N90.7, N90.8, N90.9, N91, N91.0, N91.1, N91.2, N91.3, N91.4, N91.5, N92, N92.0, N92.1, N92.2, N92.3, N92.4, N92.5, N92.6, N93, N93.0, N93.8, N93.9, N94, N94.0, N94.1, N94.2, N94.3, N94.4, N94.5, N94.6, N94.8, N94.9, N95, N95.0, N95.1, N95.2, N95.3, N95.8, N95.9, N96, N97, N97.0, N97.1, N97.2, N97.3, N97.4, N97.8, N97.9, N98, N98.0, N98.1, N98.2, N98.3, N98.8, N98.9, N99, N99.0, N99.1, N99.2, N99.3, N99.4, N99.5, N99.8, N99.9, Q60, Q60.0, Q60.1, Q60.2, Q60.3, Q60.4, Q60.5, Q60.6, Q61, Q61.0, Q61.1, Q61.2, Q61.3, Q61.4, Q61.5, Q61.8, Q61.9, Q62, Q62.0, Q62.1, Q62.2, Q62.3, Q62.4, Q62.5, Q62.6, Q62.7, Q62.8, Q63, Q63.0, Q63.1, Q63.2, Q63.3, Q63.8, Q63.9, Q64, Q64.0, Q64.1, Q64.2, Q64.3, Q64.4, Q64.5, Q64.6, Q64.7, Q64.8, Q64.9, Q65, R29.8, R30, R30.0, R30.1, R30.9, R31, R32, R33, R34, R35, R36, R39, R39.0, R39.1, R39.2, R39.8, R63.1, R93.4, R94.4 |
| **Neoplasm & Cancer** | |
|  | C00, C00.0, C00.1, C00.2, C00.3, C00.4, C00.5, C00.6, C00.8, C00.9, C01, C02, C02.0, C02.1, C02.2, C02.3, C02.4, C02.8, C02.9, C03, C03.0, C03.1, C03.9, C04, C04.0, C04.1, C04.8, C04.9, C05, C05.0, C05.1, C05.2, C05.8, C05.9, C06, C06.0, C06.1, C06.2, C06.8, C06.9, C07, C08, C08.0, C08.1, C08.8, C08.9, C09, C09.0, C09.1, C09.8, C09.9, C10, C10.0, C10.1, C10.2, C10.3, C10.4, C10.8, C10.9, C11, C11.0, C11.1, C11.2, C11.3, C11.8, C11.9, C12, C13, C13.0, C13.1, C13.2, C13.8, C13.9, C14, C14.0, C14.2, C14.8, C15, C15.0, C15.1, C15.2, C15.3, C15.4, C15.5, C15.8, C15.9, C16, C16.0, C16.1, C16.2, C16.3, C16.4, C16.5, C16.6, C16.8, C16.9, C17, C17.0, C17.1, C17.2, C17.3, C17.8, C17.9, C18, C18.0, C18.1, C18.2, C18.3, C18.4, C18.5, C18.6, C18.7, C18.8, C18.9, C19, C20, C21, C21.0, C21.1, C21.2, C21.8, C22, C22.0, C22.1, C22.2, C22.3, C22.4, C22.7, C22.9, C23, C24, C24.0, C24.1, C24.8, C24.9, C25, C25.0, C25.1, C25.2, C25.3, C25.4, C25.7, C25.8, C25.9, C26, C26.0, C26.1, C26.8, C26.9, C30, C30.0, C30.1, C31, C31.0, C31.1, C31.2, C31.3, C31.8, C31.9, C32, C32.0, C32.1, C32.2, C32.3, C32.8, C32.9, C33, C34, C34.0, C34.1, C34.2, C34.3, C34.8, C34.9, C37, C38, C38.0, C38.1, C38.2, C38.3, C38.4, C38.8, C39, C39.0, C39.8, C39.9, C40, C40.0, C40.1, C40.2, C40.3, C40.8, C40.9, C41, C41.0, C41.1, C41.2, C41.3, C41.4, C41.8, C41.9, C43, C43.0, C43.1, C43.2, C43.3, C43.4, C43.5, C43.6, C43.7, C43.8, C43.9, C44, C44.0, C44.1, C44.2, C44.3, C44.4, C44.5, C44.6, C44.7, C44.8, C44.9, C45, C45.0, C45.1, C45.2, C45.7, C45.9, C46, C46.0, C46.1, C46.2, C46.3, C46.7, C46.8, C46.9, C47, C47.0, C47.1, C47.2, C47.3, C47.4, C47.5, C47.6, C47.8, C47.9, C48, C48.0, C48.1, C48.2, C48.8, C49, C49.0, C49.1, C49.2, C49.3, C49.4, C49.5, C49.6, C49.8, C49.9, C50, C50.0, C50.1, C50.2, C50.3, C50.4, C50.5, C50.6, C50.8, C50.9, C51, C51.0, C51.1, C51.2, C51.8, C51.9, C52, C53, C53.0, C53.1, C53.8, C53.9, C54, C54.0, C54.1, C54.2, C54.3, C54.8, C54.9, C55, C56, C57, C57.0, C57.1, C57.2, C57.3, C57.4, C57.7, C57.8, C57.9, C58, C60, C60.0, C60.1, C60.2, C60.8, C60.9, C61, C62, C62.0, C62.1, C62.9, C63, C63.0, C63.1, C63.2, C63.7, C63.8, C63.9, C64, C65, C66, C67, C67.0, C67.1, C67.2, C67.3, C67.4, C67.5, C67.6, C67.7, C67.8, C67.9, C68, C68.0, C68.1, C68.8, C68.9, C69, C69.0, C69.1, C69.2, C69.3, C69.4, C69.5, C69.6, C69.8, C69.9, C70, C70.0, C70.1, C70.9, C71, C71.0, C71.1, C71.2, C71.3, C71.4, C71.5, C71.6, C71.7, C71.8, C71.9, C72, C72.0, C72.1, C72.2, C72.3, C72.4, C72.5, C72.8, C72.9, C73, C74, C74.0, C74.1, C74.9, C75, C75.0, C75.1, C75.2, C75.3, C75.4, C75.5, C75.8, C75.9, C76, C76.0, C76.1, C76.2, C76.3, C76.4, C76.5, C76.7, C76.8, C77, C77.0, C77.1, C77.2, C77.3, C77.4, C77.5, C77.8, C77.9, C78, C78.0, C78.1, C78.2, C78.3, C78.4, C78.5, C78.6, C78.7, C78.8, C79, C79.0, C79.1, C79.2, C79.3, C79.4, C79.5, C79.6, C79.7, C79.8, C79.9, C80, C80.0, C80.9, C81, C81.0, C81.1, C81.2, C81.3, C81.4, C81.7, C81.9, C82, C82.0, C82.1, C82.2, C82.3, C82.4, C82.5, C82.6, C82.7, C82.9, C83, C83.0, C83.1, C83.3, C83.5, C83.7, C83.8, C83.9, C84, C84.0, C84.1, C84.4, C84.5, C84.6, C84.7, C84.8, C84.9, C85, C85.1, C85.2, C85.7, C85.9, C86, C86.0, C86.1, C86.2, C86.3, C86.4, C86.5, C86.6, C88, C88.0, C88.2, C88.3, C88.4, C88.7, C88.9, C90, C90.0, C90.1, C90.2, C90.3, C91, C91.0, C91.1, C91.3, C91.4, C91.5, C91.6, C91.7, C91.8, C91.9, C92, C92.0, C92.1, C92.2, C92.3, C92.4, C92.5, C92.6, C92.7, C92.8, C92.9, C93, C93.0, C93.1, C93.3, C93.7, C93.9, C94, C94.0, C94.2, C94.3, C94.4, C94.6, C94.7, C95, C95.0, C95.1, C95.7, C95.9, C96, C96.0, C96.2, C96.4, C96.5, C96.6, C96.7, C96.8, C96.9, C97, D00, D00.0, D00.1, D00.2, D01, D01.0, D01.1, D01.2, D01.3, D01.4, D01.5, D01.7, D01.9, D02, D02.0, D02.1, D02.2, D02.3, D02.4, D03, D03.0, D03.1, D03.2, D03.3, D03.4, D03.5, D03.6, D03.7, D03.8, D03.9, D04, D04.0, D04.1, D04.2, D04.3, D04.4, D04.5, D04.6, D04.7, D04.8, D04.9, D05, D05.0, D05.1, D05.7, D05.9, D06, D06.0, D06.1, D06.7, D06.9, D07, D07.0, D07.1, D07.2, D07.3, D07.4, D07.5, D07.6, D09, D09.0, D09.1, D09.2, D09.3, D09.7, D09.9, D10, D37, D37.0, D37.1, D37.2, D37.3, D37.4, D37.5, D37.6, D37.7, D37.9, D38, D38.0, D38.1, D38.2, D38.3, D38.4, D38.5, D38.6, D39, D39.0, D39.1, D39.2, D39.7, D39.9, D40, D40.0, D40.1, D40.7, D40.9, D41, D41.0, D41.1, D41.2, D41.3, D41.4, D41.7, D41.9, D42, D42.0, D42.1, D42.9, D43, D43.0, D43.1, D43.2, D43.3, D43.4, D43.7, D43.9, D44, D44.0, D44.1, D44.2, D44.3, D44.4, D44.5, D44.6, D44.7, D44.8, D44.9, D45, D46, D46.0, D46.1, D46.2, D46.4, D46.5, D46.6, D46.7, D46.9, D47, D47.0, D47.1, D47.2, D47.3, D47.4, D47.5, D47.7, D47.9, D48, D48.0, D48.1, D48.2, D48.3, D48.4, D48.5, D48.6, D48.7, D48.9, E34.0, Q85.0 |
| **Respiratory diseases** | |
|  | J00, J01, J01.0, J01.1, J01.2, J01.3, J01.4, J01.8, J01.9, J02, J02.0, J02.8, J02.9, J03, J03.0, J03.8, J03.9, J04, J04.0, J04.1, J04.2, J05, J05.0, J05.1, J06, J06.0, J06.8, J06.9, J09, J10, J10.0, J10.1, J10.8, J11, J11.0, J11.1, J11.8, J12, J12.0, J12.1, J12.2, J12.3, J12.8, J12.9, J13, J14, J15, J15.0, J15.1, J15.2, J15.3, J15.4, J15.5, J15.6, J15.7, J15.8, J15.9, J16, J16.0, J16.8, J17, J17.0, J17.1, J17.2, J17.3, J17.8, J18, J18.0, J18.1, J18.2, J18.8, J18.9, J20, J20.0, J20.1, J20.2, J20.3, J20.4, J20.5, J20.6, J20.7, J20.8, J20.9, J21, J21.0, J21.1, J21.8, J21.9, J22, J30, J30.0, J30.1, J30.2, J30.3, J30.4, J31, J31.0, J31.1, J31.2, J32, J32.0, J32.1, J32.2, J32.3, J32.4, J32.8, J32.9, J33, J33.0, J33.1, J33.8, J33.9, J34, J34.0, J34.1, J34.2, J34.3, J34.8, J35, J35.0, J35.1, J35.2, J35.3, J35.8, J35.9, J36, J37, J37.0, J37.1, J38, J38.0, J38.1, J38.2, J38.3, J38.4, J38.5, J38.6, J38.7, J40, J41, J41.0, J41.1, J41.8, J42, J43, J43.0, J43.1, J43.2, J43.8, J43.9, J44, J44.0, J44.1, J44.8, J44.9, J45, J45.0, J45.1, J45.8, J45.9, J46, J47, J60, J61, J62, J62.0, J62.8, J63, J63.0, J63.1, J63.2, J63.3, J63.4, J63.5, J63.8, J64, J65, J66, J66.0, J66.1, J66.2, J66.8, J67, J67.0, J67.1, J67.2, J67.3, J67.4, J67.5, J67.6, J67.7, J67.8, J67.9, J68, J68.0, J68.1, J68.2, J68.3, J68.4, J68.8, J68.9, J69, J69.0, J69.1, J69.8, J70, J70.0, J70.1, J70.2, J70.3, J70.4, J70.8, J70.9, J80, J81, J82, J84, J84.0, J84.1, J84.8, J84.9, J85, J85.0, J85.1, J85.2, J85.3, J86, J86.0, J86.9, J90, J91, J92, J92.0, J92.9, J93, J93.0, J93.1, J93.8, J93.9, J94, J94.0, J94.1, J94.2, J94.8, J94.9, J95, J95.0, J95.1, J95.2, J95.3, J95.4, J95.5, J95.8, J95.9, J96, J96.0, J96.1, J96.9, J98, J98.0, J98.1, J98.2, J98.3, J98.4, J98.5, J98.6, J98.7, J98.8, J98.9, J99, J99.0, J99.1, J99.8, P20, P20.0, P20.1, P20.9, P21, P21.0, P21.1, P21.9, P22, P22.0, P22.1, P22.8, P22.9, Q28.9, Q30, Q30.0, Q30.1, Q30.2, Q30.3, Q30.8, Q30.9, Q31, Q31.0, Q31.1, Q31.2, Q31.3, Q31.5, Q31.8, Q31.9, Q32, Q32.0, Q32.1, Q32.2, Q32.3, Q32.4, Q33, Q33.0, Q33.1, Q33.2, Q33.3, Q33.4, Q33.5, Q33.6, Q33.8, Q33.9, Q34, Q34.0, Q34.1, Q34.8, Q34.9, R05, R06, R06.0, R06.1, R06.2, R06.3, R06.4, R06.5, R06.6, R06.7, R06.8, R07, R07.0, R07.1, R07.2, R07.3, R07.4, R09, R09.0, R09.1, R09.2 |
| **Gastrointestinal Tract Bleeding** | |
|  | K22.6, K25.0, K25.2, K25.4, K25.6, K26.0, K26.2, K26.4, K26.6, K27.0, K27.2, K27.4, K27.6, K28.0, K28.2, K28.4, K28.6, K29.0, K62.5, K66.1, K92.0, K92.1, K92.2 |

# Supplementary Table S2 – Definition of key performance healthcare indicators (KPIs)

**Supplementary Table S2.** The achievement of key performance healthcare indicators (KPIs) was considered in the calendar month prior to index date according to the reported operative definitions.

| **KPI** | **Operative definition** |
| --- | --- |
| **Effective Weekly Treatment Time** | For patients < 85 years: Effective treatment time/week ≥ 720 min; For patients ≥ 85 years: Effective treatment time/week ≥ 660 min |
| **Infusion Volume or Processed Blood Volume** | In case ≥ 75% of treatments per month done with Online HDF post dilution: Infusion volume/week ≥ 63 l (≥ 58 l if patient > 85 years); In case < 75% of treatments per month: Processed blood volume/week ≥ 240 l (≥ 220 l if patient > 85 years) |
| **Single Pool Kt/V** | [spKt/V ≥ 1.40 (at least 2 but less than 4 treatments per week)] OR  [spKt/V ≥ 1.05 (at least 4 but less than 5 treatments per week)] OR  [spKt/V ≥ 0.84 (at least 5 but less than 6 treatments per week)] OR  [spKt/V ≥ 0.70 (at least 6 treatments per week)] |
| **Vascular Access** | All treatments with fistula OR graft |
| **Hepatitis B Protection** | [Patient is between 3 and 9 months within respective clinic] OR  [Patient is not eligible (i.e., patient refused vaccination, is naturally immunized, Carrier Ag Hbs+, already vaccinated, medical decision due to patient conditions)] OR  [HbsAg positive patients] OR  [HbsAb ≥ 10 IU/l AND HbsAg NOT doubt/missing] OR  [3 HBV vaccinations done (7 months between 1st and 3rd vaccination) AND HbsAg NOT doubt/missing] |
| **Serum Albumin** | Albumin ≥ 3.5 g/dl |
| **Serum Haemoglobin** | If erythropoietin therapy (EPO) was administered or prescribed during the last 3 weeks before lab analysis date: 10.0 ≤ Haemoglobin ≤ 12 .0 g/dl; If EPO was not administered: Haemoglobin ≥ 10.0 g/dl |
| **Hydration Status (relOH)** | relOH ≤ 13% for female patients AND relOH ≤ 15% for male patients |
| **Hemodynamic Status** | If in one of the last two consecutive months:  [Pre dialysis systolic blood pressure (pre-SBP) < 130 mmHg AND without antihypertensive therapy AND with rel OH ≤ 13% for female patients and ≤ 15% for male patients] OR  [130 mmHg ≤ pre-SBP < 160 mmHg AND without antihypertensive therapy] OR  [130 mmHg ≤ pre-SBP < 160 mmHg AND with antihypertensive therapy AND with rel OH ≤ 13% for female patients and ≤ 15% for male patients]  Patient is considered with antihypertensive therapy if the drug was administered or prescribed during the month of evaluation of the KPI. |
| **Serum Phosphate** | If Phosphate Binder was administered or prescribed during the last 7 days before lab analysis date: 2.5 mg/dl ≤ Phosphate < 5.5 mg/dl; otherwise: Phosphate < 5.5 mg/dl |
| **Serum Calcium** | [8.4 ≤ Calcium corrected ≤ 10.2 mg/dl] OR  [Calcium corrected < 8.4 mg/dl after Parathyroidectomy] OR  [Calcium corrected < 8.4 mg/dl with iPTH corrected < 130 pg/ml AND with Vitamin D oral]  Patient is considered with Calcium mimetics, Vitamin D IV, Vitamin D oral if the drug was administered or prescribed during the last month before lab analysis date. |
| **Serum iPTH** | [130 ≤ iPTH corrected ≤ 585 pg/ml] OR  [iPTH corrected < 130 pg/ml after Parathyroidectomy] OR  [iPTH corrected < 130 pg/ml without Calcium mimetics, without Vitamin D IV AND 8.4 ≤ Calcium corrected ≤ 10.2 mg/dl] OR  [iPTH corrected < 130 pg/ml without Calcium mimetics, without Vitamin D IV AND Calcium corrected < 8.4 mg/dl AND with Vitamin D oral]  Patient is considered with Calcium mimetics, Vitamin D IV, Vitamin D oral if the drug was administered or prescribed during the last month before lab analysis date. |
| **Fluid Removal** | Fluid removal < 13 ml/h/Kg |
| **Erythropoietin Resistance Index (ERI)** | If patient in therapy with EPO: ERI ≤ 15 IU/kg/week/g x 100 ml |
| **Serum Ferritin** | 200 ≤ Ferritin ≤ 800 ng/ml |
| **Serum C-Reactive Protein (CRP)** | CRP ≤ 20 mg/l |
| **Lean Tissue Index (LTI)** | LTI > 10th percentile Kg/m² |
| **Serum normalized Protein Catabolic Rate (nPCR)** | PCRn > 1.1 g/Kg/day |
| **Serum Potassium (K+)** | 3.5 < K+ < 6 mEq/l |
| **Serum Bicarbonate (HCO3-)** | 18 ≤ HCO3- ≤ 26 mEq/l |
| **Serum Sodium (Na+)** | 137 < Na+ <142 mEq/l |

# Supplementary Table S3 – Definition of medications

**Supplementary Table S3.** Medication use at the index date was ascertained as the occurrence of suggestive ATC codes.

| **Medications** | **ATC Codes** |
| --- | --- |
| **Phosphate Binders** | V03AE, A12A, A02A, A02A |
| **Vitamin D and Analogues** | H05BX02, A11C |
| **Calcimimetic agents** | H05BX01 |
| **Antacids and Proton Pump Inhibitors** | A02B |
| **Anti-Inflammatory agents** | A07E, H02, M01, L04 |
| **Anti-Diabetic Drugs** | A10A, A10B, A10X |
| **Anti-thrombotic agents** | B01 |
| **Iron Supplements** | B03A |
| **Erythropoietin** | B03X |
| **Inotropic agents** | C01A, C01C |
| **Anti-Arrhythmic agents** | C01B |
| **Cardiac Vasodilators agents** | C01D |
| **Antihypertensive** | C02, C03, C04, C08, C07 |
| **Renin-angiotensin-system (RAS)-acting agents** | C09 |
| **Lipid-Modifying agents** | C10 |
| **Anticancer and Endocrine agents** | L01, L02 |
| **Xanthine Oxidase Inhibitors** | M04AA |
| **Psychotropic agents** | N03, N04, N05, N06 |
| **Bronchodilators** | R03, R06 |

# Supplementary Table S4 – Data management

**Supplementary Table S4.** A multivariate imputation by fully conditional specification regression method was applied on 31 continuous measures (SAS MI procedure) after a data cleansing step considering as missing any data that lies outside the listed upper or lower values (if indicated). Variables signed with an asterisk (*) were logit transformed before the imputation process to conform to the multivariate normality assumption of the MI procedure and transformed back to create the imputed data set.

| **Measure** | **Min** | **Max** |
| --- | --- | --- |
| Age (years) |  |  |
| Dialysis vintage (years)* | >0 |  |
| Body Mass Index (BMI) (kg/m²) | 12 | 50 |
| Number of dialysis sessions (last 30 days) |  |  |
| Treatment time (min) |  |  |
| Kt/V* | >0 |  |
| Interdialytic weight gain (IDWG) (Kg)* | >0 | 7 |
| Post-Dialysis Weight – Dry weight (Kg) | -5 | 6 |
| Pre-dialysis Systolic Blood Pressure (mmHg) |  |  |
| BIS relative Overhydration (OH/ECW)* | >0 |  |
| BIS Lean Tissue Index (LTI) (kg/m²) |  |  |
| BIS Fat Tissue Index (FTI) (kg/m²)* |  |  |
| Serum Albumin (g/dl)* |  |  |
| Serum Hemoglobin (g/dl) |  |  |
| Serum Phosphorus (mg/dl) | 1 |  |
| Serum Calcium (mg/dl) | 5.5 | 14 |
| Serum Sodium (mEq/l) | 130 | 150 |
| Serum Potassium (mEq/l)* |  |  |
| Serum iPTH (pg/ml)* | 11 | 3500 |
| Serum Bicarbonate (HCO3-) (mEq/l) |  |  |
| Serum Platelets (no./mm³)* | 15000 | 750000 |
| Serum Lymphocytes (%)* | >0 | 50 |
| Serum Neutrophils (%) | >0 | 100 |
| Serum Ferritin (ng/ml)* | 20 |  |
| Serum Transferrin Saturation (TSAT) (%)* | >0 | 100 |
| Serum C-Reactive Protein (CRP) (mg/l)* | >0 |  |
| Serum normalized Protein Catabolic Rate (nPCR) (g/Kg/day) | >0 |  |
| Serum Alanine Aminotransferase (ALT) (IU/L)* | 5 |  |
| Serum Aspartate Aminotransferase (AST) (IU/L)* | 5 |  |
| Serum LDL (mg/dl)* | 20 |  |
| Serum HDL (mg/dl)* | 10 |  |
